# Inhibition of post-translational N-glycosylation by HRD1 that controls the fate of ABCG5/8 transporter

**DOI:** 10.1038/srep04258

**Published:** 2014-03-03

**Authors:** Shingo Suzuki, Tsuyoshi Shuto, Takashi Sato, Masayuki Kaneko, Tappei Takada, Mary Ann Suico, Douglas M. Cyr, Hiroshi Suzuki, Hirofumi Kai

**Affiliations:** 1 Department of Molecular Medicine, Graduate School of Pharmaceutical Sciences, Kumamoto University, 5-1 Oe-Honmachi, Chuo-Ku, Kumamoto 862-0973, Japan; 2 Department of Medical Therapeutics and Molecular Therapeutics, Gifu Pharmaceutical University, 1-25-4 Daigaku-nishi, Gifu 501-1196, Japan; 3 Department of Pharmacy, The University of Tokyo Hospital, Faculty of Medicine, The University of Tokyo, 7-3-1 Hongo, Bunkyo-ku, Tokyo 113-8655, Japan; 4 Department of Cell and Developmental Biology, School of Medicine, 526 Taylor Hall, University of North Carolina, Chapel Hill, North Carolina 27599-7090, USA; 5 These authors contributed equally to this work.; 6 Current address: Department of Cell Fate Control, Institute of Molecular Embryology and Genetics, Kumamoto University, 2-2-1 Honjo, Kumamoto 860-0811, Japan.

## Abstract

N-glycosylation of proteins in endoplasmic reticulum is critical for protein quality control. We showed here a post-translational N-glycosylation affected by the HRD1 E3 ubiquitin ligase. Both WT- and E3-defective C329S-HRD1 decreased the level of high mannose form of ABCG8, a protein that heterodimerizes with ABCG5 to control sterol balance. Meanwhile, HRD1 increased the non-glycosylated ABCG8 regardless of its E3 activity, thereby suppressing full maturation of ABCG5/8 transporter. Pulse chase and mutational analysis indicated that HRD1 inhibits STT3B-dependent post-translational N-glycosylation of ABCG8. Whereas, HRD1 had only slight effect on the N-glycosylation status of ABCG5; rather it accelerated ABCG5 degradation in an E3 activity-dependent manner. Finally, RMA1, another E3 ubiquitin ligase, accelerated the degradation of both ABCG5 and ABCG8 *via* E3 activity-dependent manner. HRD1 and RMA1 may therefore be negative regulators of disease-associated transporter ABCG5/ABCG8. The findings also highlight the unexpected E3 activity-independent role of HRD1 in the regulation of N-glycosylation.

The secretory pathway in eukaryotic cells is accompanied by a variety of covalent modifications to the polypeptides that are newly synthesized in endoplasmic reticulum (ER)^1^. Among the modifications of secretory proteins, asparagine (N)-linked glycosylation (N-glycosylation) catalyzed by the oligosaccharyltransferase (OST) complex is one of the major modifications of both soluble and membrane-spanning proteins^2^. The N-glycans on the proteins contribute to their proper folding, assembly and stability due to their physical properties as well as to the quality control by serving as a ‘tag' for glycoproteins to be recognized by molecular chaperones, or otherwise targeted for the ER-associated degradation (ERAD)^2^. Hence, understanding how balance of protein N-linked glycosylation and de-glycosylation is regulated has been an important issue in recent years.

In general, N-glycosylation of secretory proteins is an event that occurs co-translationally. After nascent polypeptide enters ER lumen, the OST complex recognizes the sequon, Asn-X-Thr/Ser (where X can be any amino acid other than proline)^3^ and transfers the high mannose oligosaccharides co-translationally as long as the sequon is 65–75 residues away from the peptidyl-transferase site on the large ribosomal subunit^4^. STT3A, a major catalytic subunit of OST complex, is considered to be primarily responsible for the co-translational N-glycosylation of both soluble and membrane-spanning proteins^5^. Importantly, others and we recently identified the novel type of N-glycosylation that occurs post-translationally. The examples of post-translationally N-glycosylated proteins are yet limited to a few cases such as human coagulation factor VII (FVII)^6^ and excessively unfolded human transthyretin (TTR)^7^. Notably, in these cases, another STT3 isoform STT3B is considered as an important factor to mediate post-translational N-glycosylation in the OST complex.

Human ATP-binding cassette transporters (ABC transporters) are membrane transporters that use energy from ATP hydrolysis to transport a wide variety of substrates across the cellular membrane^8^. ABC transporters are classified as either full transporter containing two transmembrane domains (TMDs) and two nucleotide binding domains (NBDs) or as half transporters containing one of each domain^8^. Full transporters generally function as a monomer, while half transporters assemble as either homodimers or heterodimers to create a functional transporter. Generally, the expression level of these transporter proteins is regulated by both transcriptional and post-translational mechanisms. Among these, studies on post-translational regulation, especially N-linked glycosylation-dependent regulation, have been increasingly given attention because most of ABC transporters possess putative modification sequon for N-glycosylation^9^,^10^. However, how N-glycosylation of these multi-spanning membrane transporters is physiologically controlled and factors that are involved in the regulation are yet to be fully understood.

One of the clinically relevant ABC transporters, whose intracellular quality control system is only identified in mice^11^,^12^,^13^, is the ABCG5/ABCG8 complex, both of which are ABC half-transporters that are highly expressed in the apical membranes of small intestine and the canalicular membranes in liver. Murine ABCG5 and ABCG8 form heterodimer in ER and are expressed to the plasma membrane, where they work as a sterol transporter^11^. Defect in plasma membrane expression of ABCG5/8 complex results in a genetic disorder called sitosterolemia as well as severe premature atherosclerosis^14^,^15^, implying that monitoring the quality of ABCG5 and ABCG8 proteins is critical for the regulation of their functional expression in the cells. Although, like other ABC transporters, two and one N-glycosylation sites exist in murine ABCG5 and ABCG8, respectively^12^, the physiological relevance of N-glycosylation of human ABCG5 and ABCG8, especially the sites for N-glycosylation and the factors that regulate the N-glycosylation, are still unknown.

Here, we established expression system of human ABCG5 and ABCG8 proteins and determined the sites for N-glycosylation. Unexpectedly, the N-glycosylation sites for ABCG5 and ABCG8 were mostly post-translationally modified and STT3B is robustly involved in the post-translational N-glycosylation of ABCG8. HRD1 E3 ubiquitin ligase, regardless of its E3 ligase activity, inhibited the STT3B-dependent N-glycosylation of ABCG8, thereby destabilizing ABCG8 protein. Despite the strong effect of HRD1 on ABCG8, HRD1 accelerated ABCG5 degradation in an E3 activity-dependent manner but had only slight effect on the N-glycosylation status of ABCG5. Another E3 ubiquitin ligase RMA1 accelerated the degradation of both ABCG5 and ABCG8 without any effects on N-glycosylation status in an E3-dependent manner. Our results are the first to show an example of post-translational N-glycosylation of multi-spanning membrane glycoprotein and provide an unexpected role of HRD1 in the post-translational N-glycosylation that affects protein stability. Overall, we clearly indicate the molecular basis of HRD1- and RMA1-dependent negative regulation of disease-associated transporter ABCG5/ABCG8.

## Results

### Determination of band patterns of monomeric and heterodimeric human ABCG5 and ABCG8

To confirm the expression patterns of monomeric and heterodimeric human ABCG5 and/or ABCG8, we first expressed Myc-tagged ABCG5 (Myc-ABCG5) or HA-tagged ABCG8 (HA-ABCG8) in HEK293 cells. The isolated cell lysates were then subjected to treatment with different endoglycosidases. Both ABCG5 and ABCG8 were detected as 65-kDa and 63-kDa protein bands, respectively (Supplementary Figure S1a, lanes 1–2, 4–5; S1c,d; S2; S3). These bands were sensitive not only to PNGase F, which removes all N-linked sugars, but also to Endo H, which removes high-mannose sugars that have not yet undergone full maturation (Supplementary Figure S1c,d, non-glycosylated (Non-G) forms; S4), suggesting that the original bands derived from single expression represent ER-localized high mannose (HM) forms of ABCG5 and ABCG8 proteins, as is often the case with previous study^11^,^12^. We next sought to determine whether these molecules properly heterodimerize when coexpressed in the cells. As shown in Supplementary Figure S1a (Lane 6), S1b (lane 4), S2 and S3, coexpression of Myc-ABCG5 and HA-ABCG8 resulted in the appearance of additional high molecular weight (HMW) forms that are indicated as complex glycosylated (C-G) forms, which are considered to localize in the golgi or plasma membrane (ABCG5, 70- to 76-kDa; ABCG8, 68- to 74-kDa). Consistently, these HMW protein bands were sensitive to PNGase F, but were resistant to Endo H (Supplementary Figure S1b, lanes 5–6; S3), confirming the existence of heterodimeric human ABCG5/ABCG8 complex in coexpression condition, consistent with previous reports^11^,^12^.

### Determination of N-linked glycosylation sites in human ABCG5 and ABCG8

**Figure 1:**
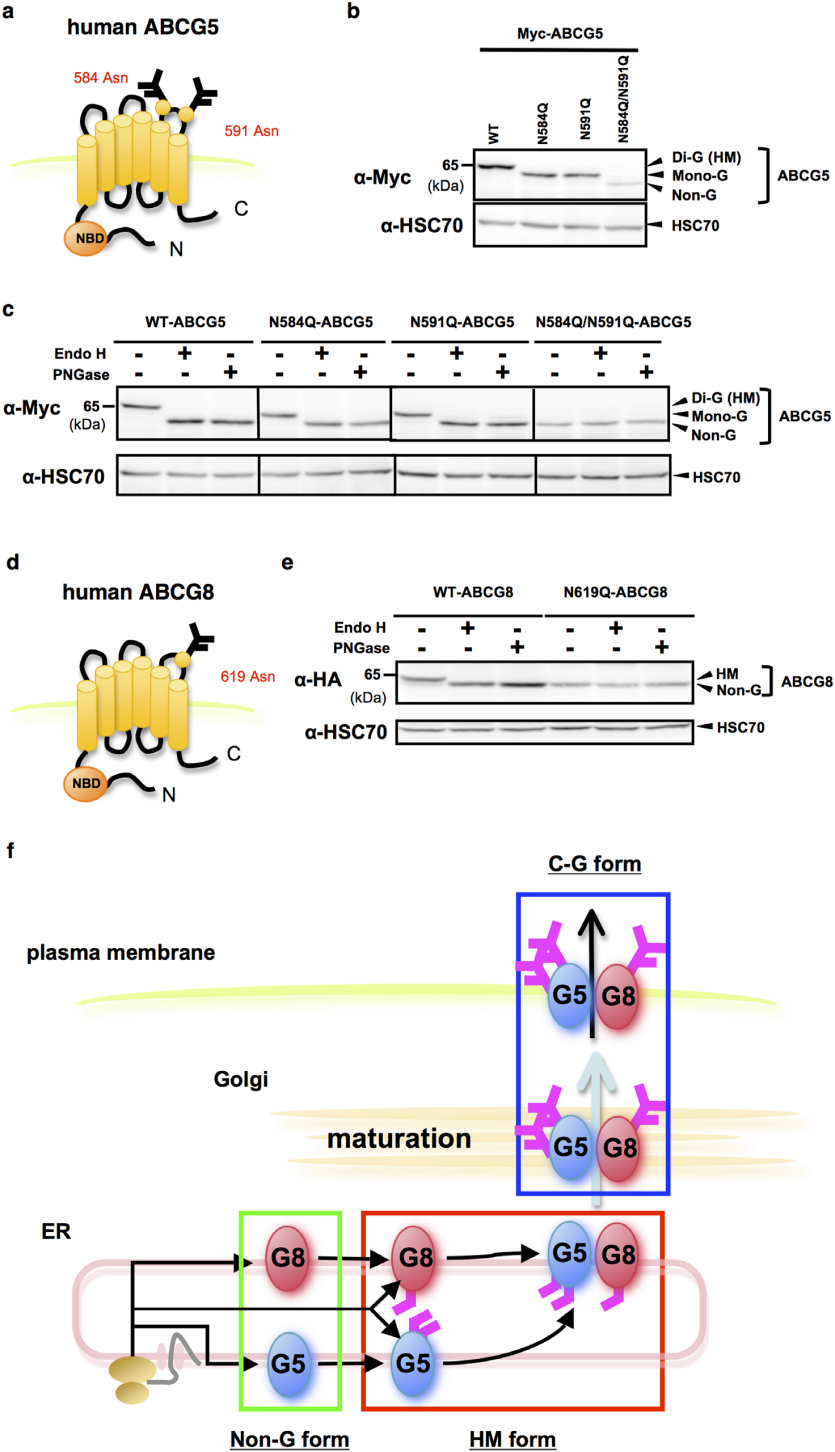
Determination of N-linked glycosylation sites in human ABCG5 and ABCG8. (a) Putative asparagine residues to be N-glycosylated in human ABCG5 protein. (b) Steady-state expression of monomeric Myc-tagged WT-, N584Q-, N591Q- and N584Q-/N591Q-ABCG5 was analyzed by immunoblotting. (c) Endo H (500 U) and PNGase F (500 U) sensitivity of Myc-tagged WT-, N584Q-, N591Q- and N584Q-/N591Q-ABCG5 proteins shown in (b). (d) Putative asparagine residue to be N-glycosylated in human ABCG8 protein. (e) Endo H and PNGase F sensitivity of HA-tagged WT- or N619Q-ABCG8 proteins. (f) Schematic flow of intracellular trafficking pathway of ABCG5 and ABCG8 proteins. Complex-glycosylated, high-mannose, di-glycosylated, mono-glycosylated and non-glycosylated forms of ABCG5 and ABCG8 proteins are represented as C-G, HM, Di-G, Mono-G and Non-G, respectively. Gels have been cropped for clarity; full-length gels of Figure 1b, 1c and 1e are presented in Supplementary Figure S4.

To determine N-glycosylation sites of human ABCG5 and ABCG8, we performed characterization of non-glycosylation mutants of these proteins. We took advantage of previous report on the N-glycosylation sites of murine ABCG5 at residue 585 and 592, and murine ABCG8 at residue 619^12^ since the sites are conserved between mice and human (Figure 1a, at residue 584 and 591 in human; 1d, at residue 619 in human). Single substitution of glutamine for asparagine at position 584 (N584Q) and at position 591 (N591Q) in human ABCG5 reduced the apparent molecular mass to a similar extent between two mutants (Figure 1b). Double mutation (N584Q/N591Q) further reduced the band size identical to those of EndoH- and PNGase F-treated ABCG5 proteins (Figure 1b,c; Supplementary Figure S4), indicating that human ABCG5 contains two N-linked glycans at positions 584 and 591. On the other hand, substitution of glutamine for asparagine at position 619 (N619Q) of human ABCG8 also reduced the molecular mass, and the band size was identical to those of EndoH- and PNGase F-treated ABCG8 proteins (Figure 1e; Supplementary Figure S4), demonstrating that human ABCG8 contains a single N-linked glycan at position 619. Moreover, the steady state expression of N-glycosylation defective mutants of human ABCG5 and ABCG8 was lower when compared to that of WT-ABCG5 and -ABCG8, respectively, suggesting that glycosylation defects in human ABCG5 and ABCG8 result in lesser protein stability (Figure 1b,e; Supplementary Figure S4).

### Co- and post-translational N-glycosylation in human ABCG5 and ABCG8

**Figure 2:**
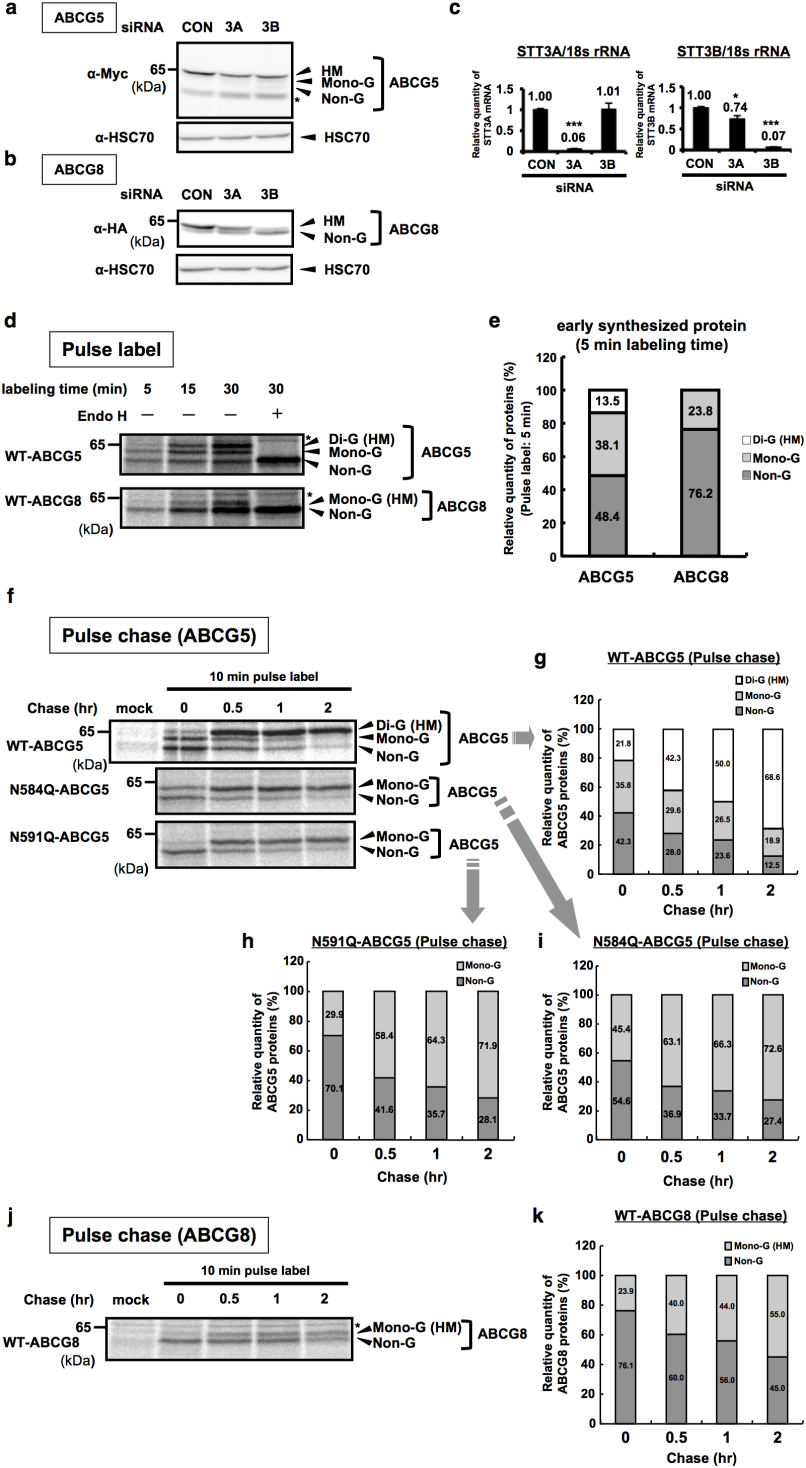
Co- and post-transcriptional N-glycosylation in human ABCG5 and ABCG8. (a), (b) STT3 siRNAs (100 nM)-transfected HEK293 cells were co-transfected with Myc-ABCG5 (a) or HA-ABCG8 (b). Cell lysates were analyzed by immunoblotting. (c) Knockdown efficiencies of STT3A and STT3B genes were analyzed by quantitative RT-PCR (Q-RT-PCR). Human 18s ribosmal RNA (18srRNA) was used as an internal control. *p < 0.05, ***p < 0.001, versus con siRNA-transfected cells; Dunnett's test (n = 3). (d), (e) Myc-ABCG5 or HA-ABCG8-transfected HEK293 cells were pulse labeled for indicated time (5, 15, 30 min) and cell lysates were harvested immediately and digested with or without EndoH. Samples were immunoprecipitated with anti-Myc or anti-HA antibody, followed by autoradiography (d). Relative quantity of each glycosylated form of ABCG5 or ABCG8 within all ABCG5 or ABCG8 bands in a short pulse-labeling period (5 min) was quantified (e). (f–i) Pulse chase (pulse labeled for 10 min) of HEK293 cells expressing Myc-tagged WT-, N584Q- and N591Q-ABCG5 (f). Relative quantity of each glycosylated form of WT-ABCG5 (g), N591Q-ABCG5 (h) and N584Q-ABCG5 (i) within all ABCG5 was quantified. (j), (k) Pulse chase of HEK293 cells expressing HA-tagged WT-ABCG8 (j) and relative quantity of each glycosylated form of WT-ABCG8 within all ABCG8 (k). Gels have been cropped for clarity; the bands were confirmed by the comparison with full-length gel images (Supplementary Figure S4) and molecular weight.

Most of N-glycosylation occurs co-translationally and is mediated by STT3A, a catalytic subunit of OST complex in mammalian cells, while rare examples of post-translational N-glycosylation mediated by STT3B-containing OST complex in mammalian cells have been reported^5^. We first examined which OST catalytic subunit is involved in the N-glycosylation of the ABCG5 and ABCG8 using STT3 isoform-specific siRNA. Immunoblotting showed that HM form of ABCG5 was decreased in cells with STT3A knockdown, while STT3B knockdown slightly decreased HM form but slightly increased Non-G form of ABCG5 (Figure 2a,c). These data suggest contribution of both STT3A and STT3B in the regulation of HM ABCG5 expression. On the other hand, HM form of ABCG8 almost disappeared but Non-G form of ABCG8 was accumulated when cells were transfected with siRNA targeting STT3B but not STT3A (Figure 2b,c). Although there is a little concern about si-STT3B specificity, the data suggest a dominant contribution of STT3B in the regulation of HM ABCG8 glycosylation. Thus, these data demonstrate that STT3A- and STT3B-containing OST complex are differentially involved in the regulation of HM ABCG5 and ABCG8 expression and glycosylation.

Next, we determined how N-glycosylation occurs in ABCG5 and ABCG8-expressing cells by pulse-label and -chase experiments. As shown in Figure 2d, three bands that correspond to non-glycosylated (Non-G), mono-glycosylated (Mono-G) and di-glycosylated (Di-G, or HM) forms of ABCG5 proteins were observed after 15 to 30 mins pulse-labeling. Similarly, two bands that correspond to Non-G and Mono-G forms of ABCG8 protein were observed after longer pulse-labeling (Figure 2d). Importantly, HM forms of both ABCG5 and ABCG8 were lower than we expected in a short pulse-labeling period (5 min) (Figure 2d,e: HM, 13.5% within all ABCG5 bands and 23.8% within all ABCG8 bands), suggesting that these proteins are originally produced as a Non-G or Mono-G forms, albeit at different degrees. The relatively higher expression levels of de-glycosylated forms of ABCG5 and ABCG8 proteins in the early synthesized proteins implies the involvement of post-translational glycosylation in the regulation of these proteins. Consistently, pulse-chase analysis using WT-ABCG5 and WT-ABCG8 revealed that the expression level of glycosylated forms of WT-ABCG5 (Di-G and Mono-G) and WT-ABCG8 (Mono-G) are gradually increased during the chase period (Figure 2f,g: 21.8% + 35.8% = 57.6% to 68.6% + 18.9% = 87.5% for WT-ABCG5; Figure 2j,k: 23.9% to 55.0% for WT-ABCG8). Based on the observed strong dependence of ABCG8 glycosylation on STT3B (Figure 2b), the robust expression of Non-G form of ABCG8 in the early synthesized protein (Figure 2d,e), and the time-dependent increase of HM form of ABCG8 (Figure 2j,k), these results suggest that ABCG8 is mainly post-translationally glycosylated possibly *via* an STT3B-dependent mechanism.

To further characterize the glycosylation timing of each Asn residues (N584 and N591) of ABCG5 protein, mutational analysis using N584Q- and N591Q-ABCG5 constructs were performed. Notably, Non-G forms of both N584Q and N591Q ABCG5 proteins were abundantly expressed (Figure 2f,h,i: 70.1% vs. 54.6%), indicating that both Asn residues (N584 and N591) have the potential to be post-translationally glycosylated although the tendency seems to be different among residues. Consistently, pulse-chase analysis using N584Q and N591Q ABCG5 revealed that the expression level of glycosylated forms of N584Q and N591Q ABCG5 are gradually increased during the chase period (Figure 2f,h,i: 45.4% to 72.6% for N584Q-ABCG5; 29.9% to 71.9% for N591Q-ABCG5). Because of the high expression of Non-G form of N591Q ABCG5 at 0-min chase (Figure 2f,h 70.1%) and of the time-dependent increase of Mono-G form of ABCG5 (Figure 2f,h), we can deduce that most of the N584 residue of ABCG5 is post-translationally glycosylated, while N591 residue is both co- and post-translationally glycosylated. The contribution of post-translational glycosylation in the ABCG5 regulation is consistent with the partial involvement of STT3B in the regulation of ABCG5 glycosylation state, which was proven by the experiment that shows a faint Non-G ABCG5 band after si-STT3B introduction (Figure 2a). Overall, the data suggest that there is a mixture of co- and post-translational mechanisms in the regulation of N-glycosylation of HM forms of ABCG5 and ABCG8 proteins although HM ABCG8 protein is mainly subjected to post-translational N-glycosylation in an STT3B-dependent manner, while HM ABCG5 N-glycosylation seems to depend on both co- and post-translational mechanisms.

### Screening of E3 ubiquitin ligases that modulate expression pattern of WT human ABCG5 or ABCG8

Despite the identification of the sites for N-glycosylation in human ABCG5 and ABCG8 proteins (Figure 1), the factors that regulate the N-glycosylation, are still unknown. Because of the fact that monomeric human ABCG5 and ABCG8 are retained in the ER as HM forms, and that proteasome is likely a major pathway for degradation of HM forms of ABCG5 and ABCG8 in ER^2^ (Supplementary Figure S5a–d), we attempted to screen ER-associated E3 ubiquitin ligases to identify the molecules involved in the regulation of expression and N-glycosylation of WT human ABCG5 or ABCG8 in ER. Screening was performed by the change of protein expression levels and patterns of HM ABCG5 and HM ABCG8 bands after the overexpression of ER-associated E3 ubiquitin ligases (WT GP78, WT RMA1, WT HRD1 and WT CHIP) that are previously shown to be involved in the degradation of several ABC transporters^16^,^17^,^18^. We also tried the overexpression of E3 activity-deficient dominant-negative mutant form of these E3 ligases (GP78 R2M, RMA1 C42S, HRD1 C329S and CHIP H260Q) in this study. Overexpression of WT or dominant negative GP78 or CHIP had little or no impact on steady state of HM forms of ABCG5 and ABCG8 (Supplementary Figure S5e,f, GP78 and CHIP). In contrast, WT RMA1, but not C42S RMA1, overexpression decreased the expression level of HM forms of ABCG5 and ABCG8 (Supplementary Figure S5e,f, RMA1), suggesting that RMA1 accelerates the degradation of both ABCG5 and ABCG8 in an E3 activity-dependent manner. Although there seems to be two bands that correspond to HM and lower molecular weight (LMW) (‡) of ABCG8 proteins in pcDNA3.1-transfected condition (Supplementary Figure S5f, pcDNA3.1), only LMW band is observed in WT RMA1-, but not C42S RMA1-transfected lanes, and these LMW bands were not altered among the samples (Supplementary Figure S5f, WT and C42S RMA1), suggesting that RMA1 specifically accelerates the degradation of HM ABCG8 band in an E3-dependent manner. On the other hand, although WT HRD1 did not affect HM form of ABCG5, C329S HRD1 strongly induced its accumulation (Supplementary Figure S5f, HRD1), implicating that HRD1 may also accelerate the degradation of ABCG5 in an E3 activity-dependent manner. Notably, both WT HRD1 and C329S HRD1 overexpression decreased the band of HM ABCG8 and increased the band of LMW of ABCG8 (#) (Supplementary Figure S5f, HRD1). Similarly, both WT HRD1 and C329S HRD1 overexpression slightly increased the bands of LMW of ABCG5 (†) (Supplementary Figure S5e, HRD1). Taken together, although the effect of HRD1 on the regulation of HM ABCG8 protein and the characteristics of LMW bands of ABCG5 (†) and ABCG8 (‡, #) are yet unclear, the data indicate that RMA1 and HRD1 are the critical molecules that control the expression of both ABCG5 and ABCG8 proteins.

### HRD1 accumulates non-glycosylated human ABCG8

**Figure 3:**
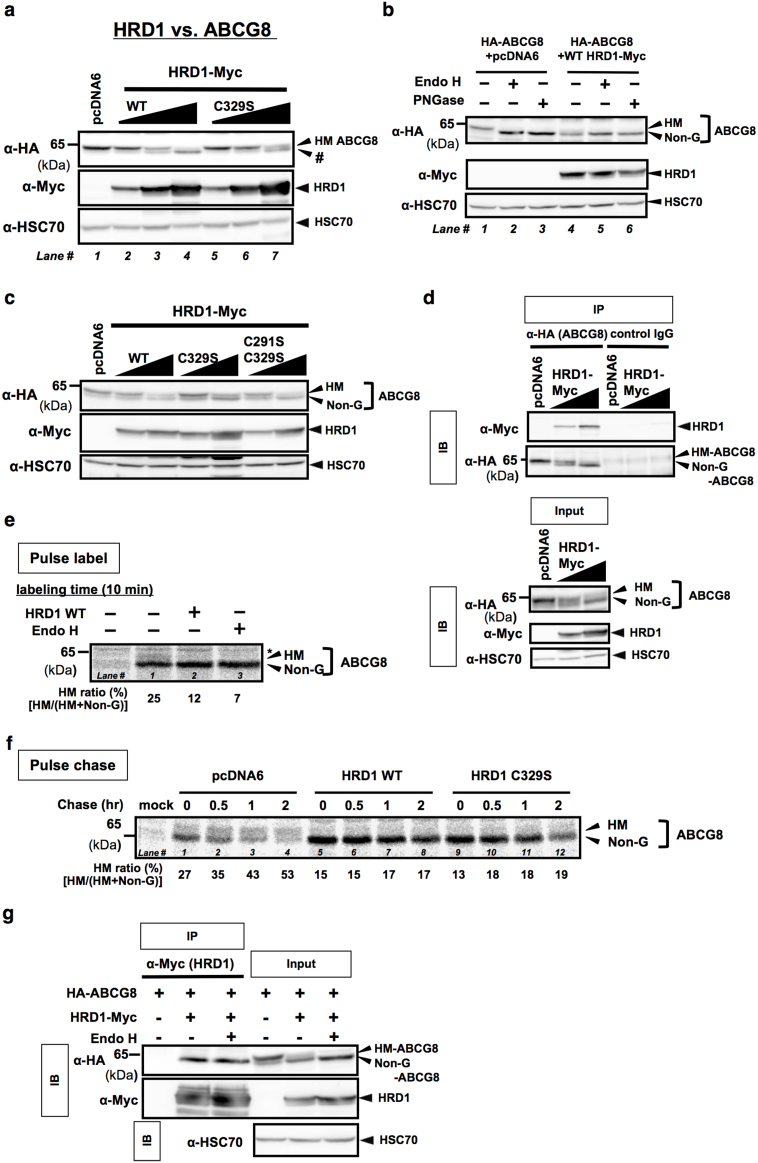
HRD1 accumulates non-glycosylated human ABCG8 by inhibiting STT3B-dependent post-translational N-glycosylation. (a–c) Cell lysates from HA-ABCG8-expressing HEK293 cells transfected with empty vector or serial amounts of expression vectors that encode Myc-tagged WT or C329S HRD1 (a), (c) or C291S/C329S HRD1 (c) were analyzed by immunoblotting. # indicates the band of LMW. Cell lysates from WT HRD1-transfected HEK293 cells were digested with EndoH or PNGase F, and analyzed by immunoblotting (b). (d) HEK293 cells were transiently co-transfected with HA-ABCG8 and Myc-HRD1. ABCG8 protein was immunoprecipitated by using anti-HA antibody or non-immunized IgG (control IgG) and immunoprecipitants were analyzed by Western blotting (upper panels). Input protein, cell lystes without immunoprecipitation of antibodies, was used as a positive control (lower panels). (e) HA-tagged ABCG8-expressing HEK293 cells were transfected with WT-HRD-1, and pulse labeled for 10 min. Cell lysates were harvested immediately and were digested with or without EndoH, and immunoprecipitated with anti-HA antibody, followed by autoradiography. HM ratio (%) was calculated based on the band intensities of HM and Non-G ABCG8 proteins [HM/(HM + Non-G)]. (f) Pulse chase analysis of HA-ABCG8-expressing HEK293 cells transfected with empty vector, Myc-tagged WT HRD-1 or C329S HRD1. After the indicated chase period, cell lysates were immunoprecipitated with anti-Myc antibody, followed by analysis with autoradiography. HM ratio (%) was calculated as explained in (e). (g) Samples in (d) were digested with or without EndoH and immunoprecipitated with HRD1 protein using anti-Myc antibody or control IgG and immunoprecipitants were analyzed by Western blotting. Input protein (cell lystes without immunoprecipitation of antibodies) was used as positive control. Gels have been cropped for clarity; the bands were confirmed by the comparison with full-length gel images (Supplementary Figure S4) and molecular weight.

We next sought to determine the nature LMW band (#) that was significantly observed when cells were co-transfected with ABCG8 and WT or C329S HRD1. We further confirmed the increased expression of LMW band by the dose-dependent transfection with WT HRD1 (Figure 3a, lanes 1–4). Because the decrease in molecular weight was 2–3 kDa and human ABCG8 possesses one site for N-glycosylation (Figure 1d,e), we hypothesized that the LMW band (#) is Non-G form of human ABCG8. As expected, HRD1-induced LMW of human ABCG8 was similar in size to Endo H- or PNGase F-treated human ABCG8 (Non-G) (Figure 3b, lanes 2–4). Moreover, Endo H or PNGase F had no impact on the size of HRD1-induced LMW band (Figure 3b, lanes 4–6), indicating that HRD1-induced LMW band was indeed the Non-G form of human ABCG8. Notably, effect of dominant-negative mutant form of HRD1 (C329S HRD1) was identical to that of WT form (Figure 3a, lanes 1, 5–7), suggesting that HRD1-induced LMW band appearance does not require its E3 activity. This was also confirmed by another dominant-negative mutant form of HRD1 (C291S/C329S HRD1) (Figure 3c). In these conditions, ABCG8 protein can interact with WT HRD1 (Figure 3d), suggesting that HRD1 overexpression decreases N-glycosylated human ABCG8 and accumulates non-glycosylated ABCG8 possibly through their interaction.

### HRD1 inhibits STT3B-dependent posttranslational N-glycosylation of human ABCG8

We further investigated whether HRD1-induced accumulation of Non-G form of human ABCG8 was due to the inhibition of N-glycosylation by HRD1. Because, as shown in Figure 2, most of ABCG8 protein is likely post-translationally N-glycosylated by STT3B, we next sought to understand the possible involvement of HRD1 on the regulation of posttranslational N-glycosylation. Pulse chase analysis after HRD1 transfection was performed and showed that when pulse labeled for 10 min, a tiny HM band can be observed (Figure 3e, lane 1, 25%) and this band is diminished by WT HRD1 expression (Figure 3e, lane 2, 12%). Moreover, despite the noticeable conversion from Non-G to HM form during 2 hrs chase period (Figure 3f, lanes 1–4, from 27 to 53%), conversion was inhibited in both WT and C329S HRD1-transfected conditions (Figure 3f, lanes 5–8, from 15 to 17%; lanes 9–12, from 13 to 19%). Finally, co-immunoprecipitation assay revealed that non-glycosylated, but not glycosylated, human ABCG8 interacted with WT HRD1 (Figure 3g), suggesting that HRD1 inhibits posttranslational N-glycosylation of human ABCG8 possibly by interacting with ABCG8 protein before sugar chains are added by STT3B-containing OST complex.

### Characterization of HRD1 regions responsible for the accumulation of non-glycosylated human ABCG8

**Figure 4:**
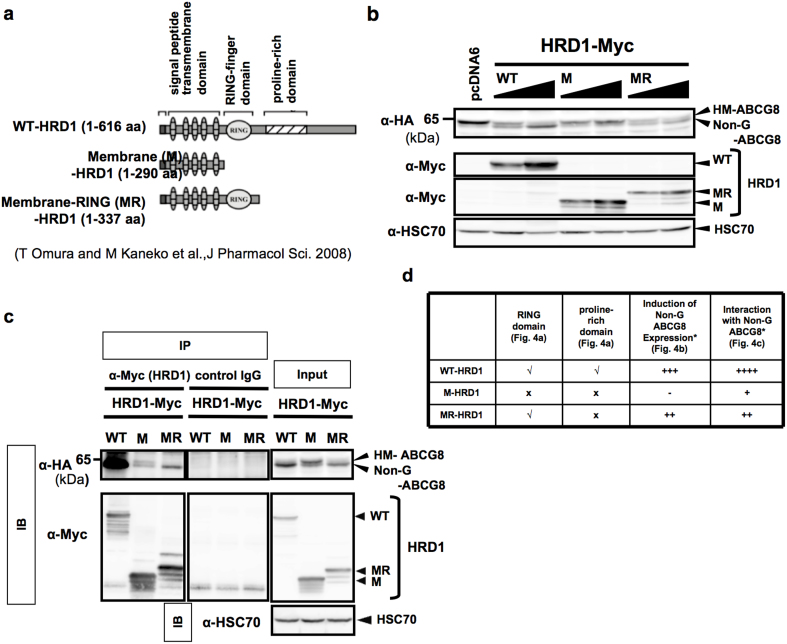
RING finger domain is critical for HRD1-induced accumulation of non-glycosylated human ABCG8. (a) Schematic representation of the HRD1 constructs. The panel diagrammatically represents WT-HRD1 and a variety of deletion mutants-HRD1 (M- and MR-HRD1). Numbers in parentheses indicate the corresponding amino acid residues of HRD1. (b) HEK293 cells were co-transfected with HA-ABCG8 and WT-, M- or MR-HRD1. Cell lysates were recovered 48 hr after transfection, followed by immunoblotting. HSC70 was used as internal control. (c) HEK293 cells were co-transfected with HA-ABCG8 plasmid and expression vectors that encode Myc-tagged WT-, M- or MR-HRD1. HRD1 protein was immunoprecipitated by using anti-Myc antibody or control IgG, and immunoprecipitants were analyzed by immunoblotting. Input protein was used as positive control. (d) Summary of the effects of WT-, M- and MR-HRD1 on the induction of non-G ABCG8 expression based on the analysis as shown in (b), (c). A tick indicates the presence of the domains whereas a cross indicates the absence of the domains in each protein structure. The levels of Non-G ABCG8 induction and the strength of interaction with Non-G ABCG8 were indicated as a different number of plus signs (+) in each column. Gels have been cropped for clarity; the bands were confirmed by the comparison with full-length gel images (Supplementary Figure S4) and molecular weight.

To investigate which HRD1 region inhibits N-glycosylation of human ABCG8, a series of HRD1 mutants was prepared (Figure 4a). HEK 293 cells were transiently transfected with human ABCG8 along with WT HRD1, membrane (M) HRD1 or membrane-RING (MR) HRD1. WT and MR but not M HRD1 induced accumulation of Non-G form of human ABCG8 (Figure 4b). Importantly, immunoprecipitation analysis further revealed a strong interaction between WT HRD1 and Non-G ABCG8 proteins (Figure 4d). Notably, there is also an interaction between MR HRD1, but not M HRD1, and Non-G ABCG8 proteins (Figure 4c,d). Moreover, MR HRD1, but not M HRD1, induced Non-G ABCG8 expression (Figure 4b,d). Since the levels of induction of Non-G ABCG8 expression and of interaction with Non-G ABCG8 are positively correlated (Figure 4d), HRD1-dependent Non-G ABCG8 induction depends on the strength of their interaction. Because of the facts that both WT and MR HRD1 proteins possess RING domain and proline-rich domain is not included in MR HRD1, RING finger domain of HRD1 is likely a critical region in both its interaction with Non-G ABCG8 and its induction of Non-G ABCG8 expression, while proline-rich domain of HRD1 may partially contribute to its binding to Non-G ABCG8 protein that increases induction of Non-G ABCG8 expression.

### ABCG8 N-glycosylation is critical for its protein stability and HRD1 accelerates ABCG8 degradation by diminishing ABCG8 N-glycosylation

**Figure 5:**
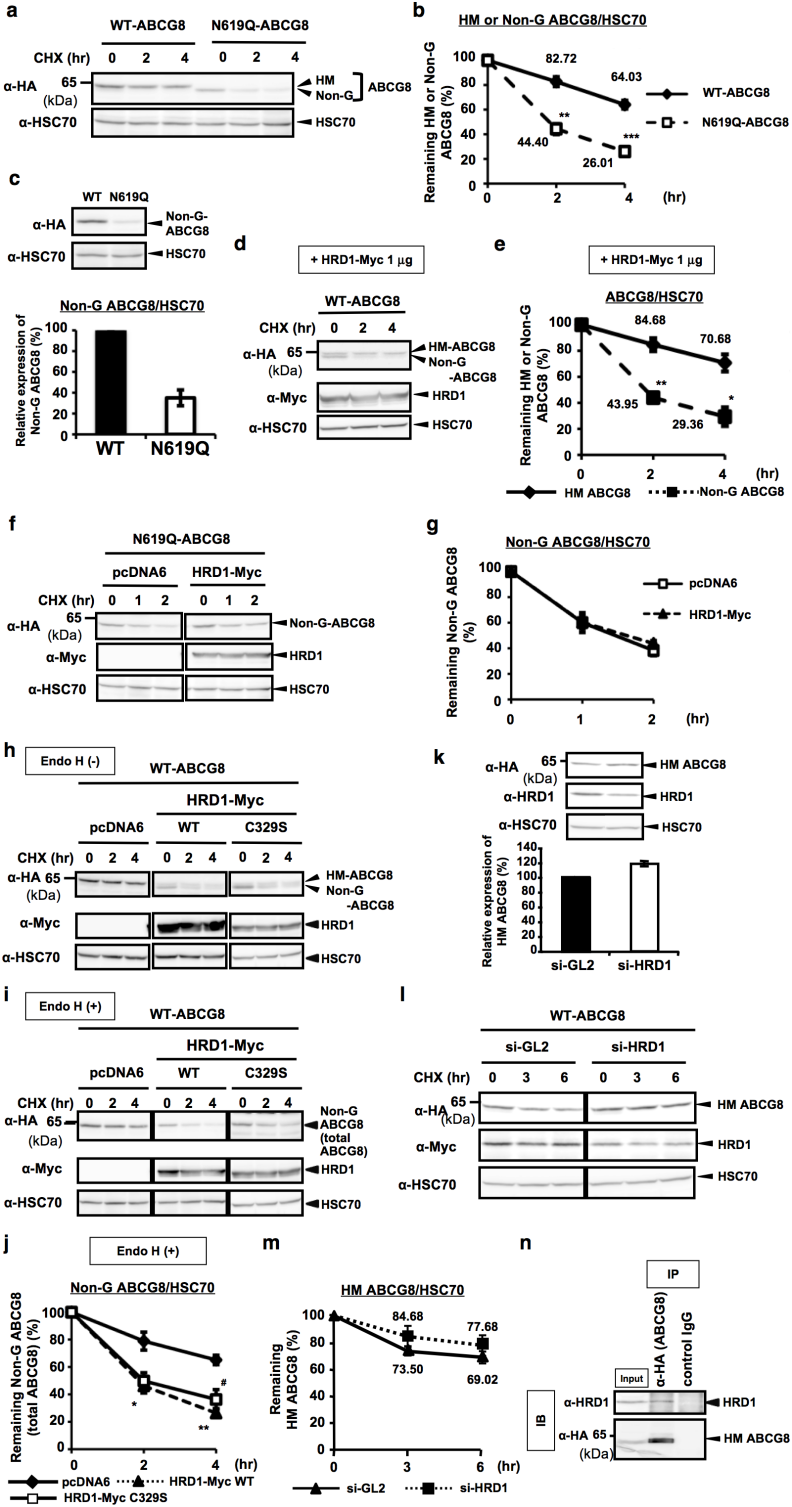
HRD1 accelerates ABCG8 degradation by reducing its protein stability through the inhibition of ABCG8 N-glycosylation. (a), (b) Stability of WT- or N619Q-ABCG8 in HEK293 cells was determined by CHX (50 μM) chase experiment (a). HM or Non-G forms of ABCG8 were quantified and normalized by HSC70 expression. Data are presented as the percentage of the amount detected at 0 hr (b). **p < 0.01, ***p < 0.001, versus WT ABCG8-transfected cells; Student's t test (n = 3). (c) Steady-state expression of HEK293 cells transfected with WT- or N619Q-ABCG8. Cell lysates were subjected to immunoblotting (upper panel). Relative expression of Non-G form of ABCG8 were calculated (n = 3) (lower panel). (d), (e) Stabilities of HM and Non-G forms of WT-ABCG8 protein derived from Myc-HRD1 (1 μg)-transfected HEK293 cells were determined by CHX chase experiment (d). HM or Non-G forms of WT-ABCG8 were quantified (e). *p < 0.05, **p < 0.01, versus HM ABCG8 bands; Student's t test (n = 3). (f), (g) Stability of Non-G N619Q-ABCG8 protein in HEK293 cells transfected with Myc-HRD1 (f). Non-G N619Q-ABCG8 protein was quantified (n = 3) (g). (h–j) WT-ABCG8 and Myc-tagged WT- or C329S-HRD1 were co-transfected and cells are subjected to CHX chase experiment. After recovery, cell lysates were digested with (i) or without (h) EndoH, and were analyzed by immunoblotting. Stability of total ABCG8 protein after transfection with HRD1 constructs was quantified using Endo H (+) shown in (i) and normalized by HSC70 expression (j). *p < 0.05, **p < 0.01, ^#^p < 0.05, versus pcDNA6-transfected cells; Student's t test (n = 3). Student's t test (n = 3). (k) Steady-state expression of HEK293 cells transfected with si-GL2 (control si-RNA) or si-HRD1. Cell lysates were subjected to immunoblotting (upper panel). Relative expression of HM ABCG8 was calculated (n = 3) (lower panel). (l), (m) Stabilities of HM form of WT-ABCG8 protein derived from si-GL2 or si-HRD1-transfected HEK293 cells (l). HM form of WT-ABCG8 was quantified (m) (n = 6 for si-GL2, n = 3 for si-HRD1). (n) ABCG8 protein transfected in HEK293 cells was immunoprecipitated and immunoprecipitants were analyzed by Western blotting (IB). Input protein, cell lystes without immunoprecipitation of antibodies, was used as a positive control (input). Gels have been cropped for clarity; the bands were confirmed by the comparison with full-length gel images (Supplementary Figure S4) and molecular weight.

As shown in Figure 1e, because the steady state expression of N-glycosylation defective mutant of human ABCG8 was lower when compared to that of WT-ABCG8, we sought to evaluate the protein stability of human N619Q ABCG8 in detail. As shown in Figure 5a and 5b, protein stability of N619Q ABCG8 was clearly lower than that of WT ABCG8. Steady state expression of N619Q further indicated lower protein stability of human N619Q ABCG8 (Figure 5c). To determine whether the stability of WT HRD1-induced Non-G form of ABCG8 is also decreased, we transfected HEK293 cells with 1 μg of HRD1 construct, which resulted in the appearance of both Non-G and HM forms of ABCG8, and CHX chase experiments were performed. Importantly, protein stability of Non-G form of ABCG8 derived from HRD1 transfection was also lower than that of HM form of ABCG8 (Figure 5d and 5e). Percentage of remaining Non-G form derived from HRD1-transfected ABCG8 protein at 2 and 4 hrs was almost identical to that of N619Q ABCG8 (Figure 5b,e, 44.40% vs. 43.95% at 2 hrs; 26.01% vs. 29.36% at 4 hrs). Moreover, the percentage of remaining HM form at 2 and 4 hrs was also identical to that of WT ABCG8 (Figure 5b and 5e, 82.72% vs. 84.68% at 2 hrs; 64.03% vs 70.68% at 4 hrs). These data suggest that HRD1 does not directly affect protein stabilities of Non-G and HM forms of ABCG8 proteins *per se*. Consistently, HRD1 does not further affect protein stability of Non-G N619Q ABCG8 protein (Figure 5f,g).

Having demonstrated that WT and C329S HRD1 induce the accumulation of Non-G ABCG8 protein that has a lower stability than HM ABCG8 protein, these HRD1 proteins may accelerate ABCG8 degradation by increasing Non-G form of ABCG8 protein. We transfected HEK293 cells with 2 μg of HRD1 constructs, which resulted in the appearance of only Non-G form of ABCG8, and CHX chase experiments were performed (Figure 5h). The cell lysates were further digested with Endo H to precisely quantify total amount of ABCG8 protein (Figure 5i,j). As we expected, similar decreased protein stabilities of Non-G forms derived from WT HRD1- and C329S HRD1-coexpressed ABCG8 were observed (Figure 5h–j), indicating that HRD1 certainly decreases total ABCG8 protein stability in the E3 activity-independent manner. To reveal that endogenous HRD1 also affects ABCG8 protein expression, we decrease HRD1 expression by siRNA technique. Importantly, knockdown of HRD1 increases the steady state expression of ABCG8 protein (Figure 5k), suggesting that endogenous HRD1 suppresses ABCG8 protein expression. However, si-HRD1-dependent stabilization of HM-ABCG8 protein *per se* was relatively slight (Figure 5l, m). In this condition, ABCG8 protein interacts with endogenous HRD1 (Figure 5n). Due to an undetectable base line expression of Non-G ABCG8 protein in our analysis, the si-HRD1 experiment could not directly demonstrate that endogenous, in addition to exogenous, HRD1 proteins inhibit N-glycosylation of HM ABCG8. Overall, our data support the idea that ABCG8 N-glycosylation is critical for its protein stability and HRD1 lessens ABCG8 expression, conceivably by diminishing ABCG8 N-glycosylation.

### RMA1 facilitates ERAD of HM forms of human ABCG5 and ABCG8 proteins

**Figure 6:**
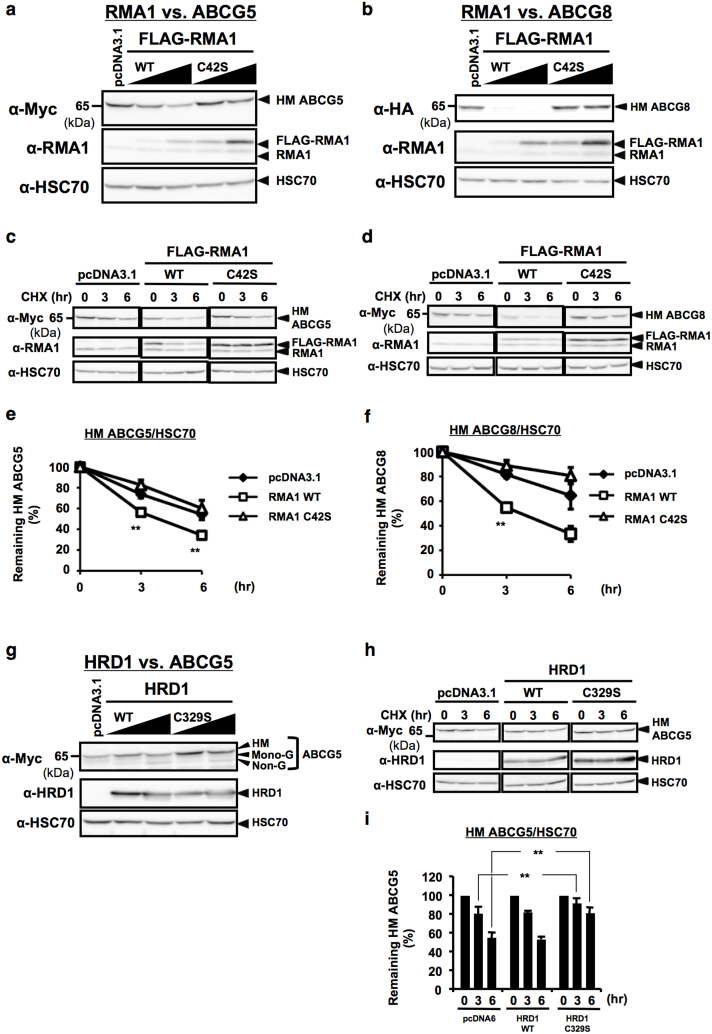
RMA1 and/or HRD1 facilitates ERAD of HM forms of human ABCG5 and/or ABCG8 proteins. (a), (b) Steady-state expression of ABCG5 (a) and ABCG8 (b) proteins in HEK293 cells transfected with empty vector or serial amounts of expression vectors that encode Flag-tagged WT or C42S RMA1 were analyzed by immunoblotting. (c–f) Stability of ABCG5 (c) or ABCG8 (d) proteins in HEK293 cells transfected with empty vector, WT or C42S RMA1 was determined by CHX (50 μM) chase experiments. HM forms of ABCG5 (e) or ABCG8 (f) were quantified and normalized by HSC70 expression. Data are presented as the percentage of the amount detected at 0 hr. **p < 0.01, versus pcDNA3.1-transfected cells; Student's t test (n = 3–6). (g) Steady-state expression of ABCG5 protein in HEK293 cells transfected with empty vector or serial amounts of expression vectors that encode WT or C329S HRD1 were analyzed by immunoblotting. (h), (i) Stability of ABCG5 protein in HEK293 cells transfected with empty vector, WT or C329S HRD1 was determined by CHX (50 μM) chase experiments (h). HM form of ABCG5 was quantified and normalized by HSC70 expression. Data are presented as the percentage of the amount detected at 0 hr (i). **p < 0.01, versus pcDNA6-transfected cells; Student's t test (n = 6). (j) Schematic flow of E3 activity-dependent posttranslational regulation of ABCG5 and ABCG8 by RMA1 and HRD1. RMA1 accelerates the degradation of both ABCG5 and ABCG8 proteins, and HRD1 also seems to accelerate the degradation of ABCG5 protein, in an E3 activity-dependent manner. Gels have been cropped for clarity; the bands were confirmed by the comparison with full-length gel images (Supplementary Figure S4) and molecular weight.

Having suggested that WT RMA1 overexpression strongly decreases both HM forms of human ABCG5 and ABCG8 proteins (Supplementary Figure S5e,f), we further determined the relationship between RMA1 and HM form of ABCG5 or ABCG8. Similar to Supplementary Figure S5, steady states of HM forms of human ABCG5 and ABCG8 proteins were reduced by WT RMA1 overexpression, but not by C42S RMA1 (Figure 6a,b). Consistently, CHX chase analysis further confirmed that only WT RMA1 accelerates degradation of HM form of ABCG5 and ABCG8 proteins, while C42S RMA1 does not seem to induce degradation (Figure 6c–f). In these conditions, HM forms of ABCG5 and ABCG8 proteins interact with WT RMA1 as well as C42S RMA1 (Supplementary Figure S6a,b). These data clarify that RMA1 suppresses the expression of both monomeric human ABCG5 and ABCG8 proteins probably through its E3 activity.

### HRD1 mainly facilitates ERAD of HM forms of human ABCG5 proteins

**Figure 7:**
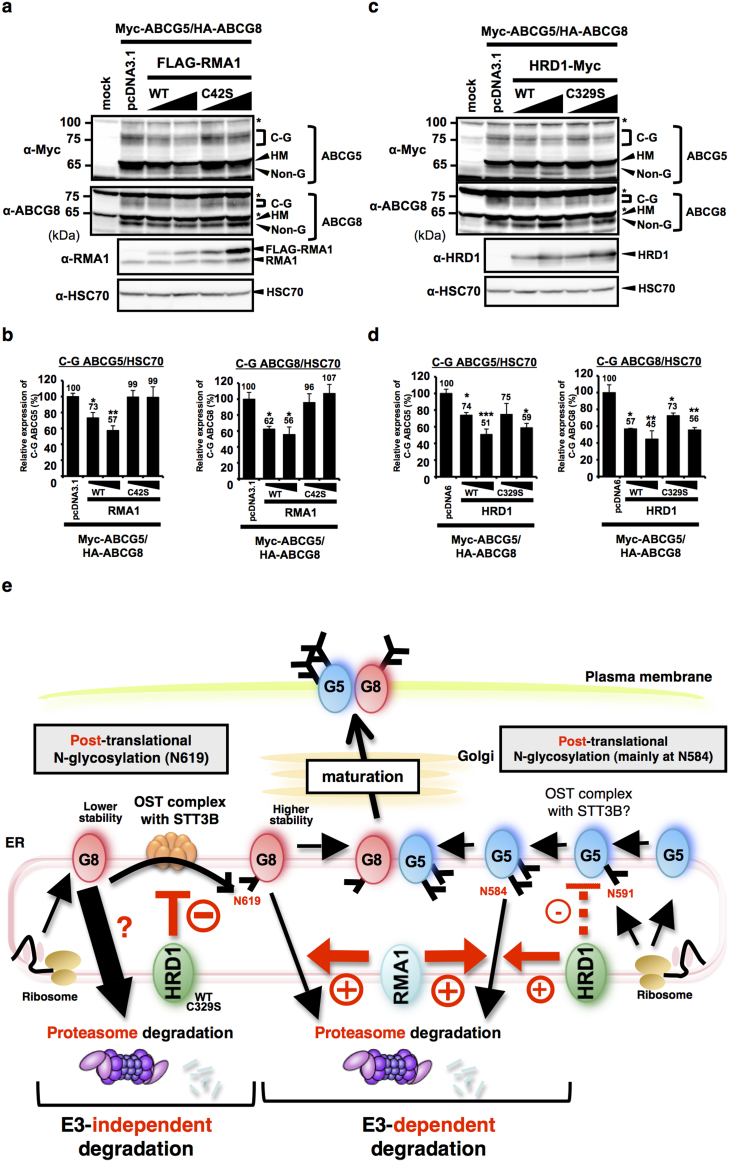
RMA1 and HRD1 are negative regulators of ABCG5/G8 maturation. (a–d) Steady-state expression of C-G forms of ABCG5 and ABCG8 proteins in HEK293 cells transfected with empty vector or serial amounts of expression vectors that encode Flag-tagged WT or C42S RMA1 (a) and Myc-tagged WT or C329S HRD1 (c) was analyzed by immunoblotting. * indicates non-specific band. Mature C-G forms of ABCG5 and ABCG8 protein bands after transfection with RMA1 (b) and HRD1 (d) constructs were calculated and data are presented as the percentage of the amount detected in empty vectors-transfected control. *p < 0.05, **p < 0.01, ***p < 0.001, versus empty vector-transfected cells; Dunnett's test (n = 3). Gels have been cropped for clarity; the bands were confirmed by the comparison with full-length gel images (Supplementary Figure S2 and S3) and molecular weight. (e) Schematic flow of RMA1- and HRD1-dependent post-translational regulation of ABCG5 and ABCG8 proteins. RMA1 accelerates the degradation of both ABCG5 and ABCG8 proteins, and HRD1 mainly accelerates the degradation of ABCG5 protein, in an E3 activity-dependent manner, while HRD1 accelerates ABCG8 degradation by diminishing the STT3B-dependent post-translational N-glycosylation of ABCG8 in an E3 activity-independent manner. Since ABCG5 N-glycosylation is also mediated by post-translational mechanism (mainly at N584 and partially at N591), HRD1 may inhibit ABCG5 N-glycosylation although the possibility seems to be minor based on the data. Hence, both RMA1 and HRD1 are negative regulators of ABCG5/ABCG8 maturation.

We also confirmed the effect of HRD1 on HM form of ABCG5. Similar to Supplementary Figure S5, WT HRD1 did not affect HM form of ABCG5, while C329S HRD1 strongly induced its accumulation (Figure 6g). Consistently, CHX chase analysis also revealed that C329S HRD1 strongly attenuates protein degradation of HM form of ABCG5 protein during chase period (Figure 6h,i). In these conditions, HM form of ABCG5 protein interacts with WT HRD1 as well as C329S HRD1 (Supplementary Figure S6c). On the other hand, although both WT HRD1 and C329S HRD1 overexpression slightly increased the band of LMW of ABCG5, which is considered as the Non-G ABCG5 protein (Figure 6g, Non-G; Supplementary Figure S7, lanes 4–6, Non-G) as it is also observed in Supplementary Figure S5e (†), induction of Non-G ABCG5 was much less than that observed in the case of Non-G ABCG8 protein. Overall, these data demonstrate that RMA1 accelerate the degradation of both ABCG5 and ABCG8 proteins, and HRD1 seems to mainly accelerate the degradation of ABCG5 protein, in an E3 activity-dependent manner (Figure 7e).

### The effect of RMA1 or HRD1 on maturation of ABCG5/G8 complex

Finally, to determine the role of RMA1 and HRD1 in the regulation of plasma membrane expression of ABCG5/ABCG8 complex, we transfected the cells with both ABCG5 and ABCG8 constructs together with WT or E3 defective mutants of RMA1 or HRD1. As determined in Figure S1A and S1B, C-G forms of ABCG5 and ABCG8 after various combination of transfection were quantified by immunoblot gel images. Consistent with the effect of WT and C42S RMA1 on monomeric expression of ABCG5 and ABCG8, only WT but not C42S RMA1 reduced the expression of C-G forms of ABCG5 and ABCG8 (Figure 7a,b), suggesting E3 activity-dependent negative regulatory role of RMA1 in the posttranslational regulation of ABCG5/ABCG8 complex. On the other hand, both WT and C329S HRD1 reduced expression of C-G forms of ABCG5 and ABCG8 to an almost similar pattern and extent (Figure 7c,d), indicating E3 activity-independent negative regulatory role of HRD1 in the posttranslational regulation of ABCG5/ABCG8 complex.

## Discussion

N-glycosylation is one of the important post-translational modifications that occur in the ER^2^. Our study focuses on identification of mechanisms responsible for N-glycosylation of ABCG5/8 transporters, and of factors involved in its regulation. Based on our results, the following model is proposed for N-glycosylation-associated quality control of ABCG5 and ABCG8 proteins by the E3 ligase proteins in the ER (Figure 7e). Among two E3 ligase proteins determined by overexpression-based screening, RMA1 is likely a factor that accelerates the degradation of both ABCG5 and ABCG8 proteins in an E3 activity-dependent manner. HRD1, another E3 ligase protein, also mainly accelerates the degradation of ABCG5 protein probably through E3 activity-dependent mechanism. Having demonstrated that RMA1 and HRD1 are well-established E3 ubiquitin ligases and their RING finger domains exert suppressive effects through their capability to induce ERAD^16^,^17^, it is highly likely that the effects are E3 activity-dependent although direct ubiquitin addition was not monitored in the present study. In contrast, HRD1 does not directly accelerate the degradation of ABCG8 protein, but rather accelerates its degradation through an indirect mechanism, that is, diminished STT3B-dependent post-translational N-glycosylation of ABCG8, which lessens the stability of ABCG8 protein. This process may be designated as “E3-independent degradation” (Figure 7e) because of the fact that E3 activity-defective mutant of HRD1 (C329S and C329S/C291S) could also increase Non-G form of ABCG8 (Figure 3a,c). On the other hand, HRD1 has only slight effect on the N-glycosylation of ABCG5, although post-translational mechanism is also involved in the N-glycosylation at Asn 584 of ABCG5, and partly involved at Asn 591 of ABCG5. Given the facts that (1) RING domain-containing MR HRD1, but not RING domain-missing M HRD1, could bind to ABCG8 and induce Non-G form of ABCG8 (Figure 4), (2) interaction of HRD1 with ABCG8 protein was limited to its Non-G form (Figure 3h), and (3) the conversion from Non-G to HM forms of ABCG8 was inhibited in both WT and C329S HRD1-transfected conditions (Figure 3f), HRD1 exerts its inhibitory action probably through its RING finger domain. This is the first report to show that RING-finger domain in E3 ubiquitin ligases contributes to protein-protein interaction without directly affecting the proteasomal degradation pathway, which leads to diminished N-glycosylation of substrate by STT3B. Overall, it is quite possible that HRD1 masks the N-glycosylation site of ABCG8 to protect from STT3B-dependent glycosylation.

One of the key findings in this study is that both ABCG5 and ABCG8 are post-translationally N-glycosylated. Our mutational metabolic labeling analysis further suggested that ABCG8 is post-translationally N-glycosylated at N619 in an STT3B-dependent manner, while ABCG5 is N-glycosylated depending on both co- and post-translational mechanisms at N584 and N594 although N584 tends to be more post-translationally glycosylated based on the pulse-chase analysis (Figure 2f,h,i). What determines the timing of glycosylation, co- or post-translational modification, and STT3s' enzymatic specificities? Importantly, C-terminal Asn-X-Thr sites, but not Asn-X-Ser sites, located at <50 residues (boundary is located between 50 and 55 residues from the C terminus of a protein) appear to be preferably post-translationally N-glycosylated more efficiently by STT3B in other substrate proteins^19^. Notably, sequons locate at 54–67 residues of ABCG5 and ABCG8 proteins, implying that those sites have a potential to be post-translationally N-glycosylated. In fact, the expression analysis of Non-G forms of Asn-mutated ABCG5 and WT-ABCG8 proteins during pulse period indicated that all Asn sites (N584 and N591 of ABCG5 and N619 of ABCG8) are post-translationally N-glycosylated, albeit at different levels (N619 of ABCG8 ≥ N584 of ABCG5 > N591 of ABCG5). Consistently, the sequons of the highly probable sites, N619 of ABCG8 and N584 of ABCG5, are Asn-Leu-Thr and Asn-Phe-Thr, respectively, both of which fits the above-mentioned rule of post-translational N-glycosylation in terms of amino acid sequences (Asn-X-Thr rule) (Supplementary Figure S8). Together, these data are consistent with the finding that shows relationship between sequon and timing of glycosylation (co- or post-translational) shown by Shrimal et al.^19^. To make sure this rule can be also applied for other ABCG subfamily members, we performed sequon analysis in other ABCG family proteins. As shown in Supplementary Figure S5, in addition to ABCG5 and ABCG8, sequon of ABCG2 (BCRP) protein around position 596 is Asn-Ala-Thr and sequon locates at 59 residue of ABCG2 protein, implicating that ABCG2 could be also post-translationally N-glycosylated. Despite an absence of direct evidence that supports this hypothesis, our previous study showed that overexpression of HRD1, which is shown to strongly inhibit post-translational N-glycosylation of ABCG8 in the present study, also slightly increases Non-G form of ABCG2 (Figure 1D in the report)^20^, supporting the idea that the N-glycosylation of ABCG2 is mediated by post-translational mechanism in which STT3B and HRD1 may be involved. Further pulse chase analysis would be needed to confirm this hypothesis.

Despite the clear evidence of the contribution of STT3B to the post-translational N-glycosylation of ABCG8 based on the si-STT3B analysis (Figure 2b), which STT3 isoform contributes to the post-translational N-glycosylation of ABCG5 could not be clearly identified in the present study. Since STT3B knockdown slightly accumulates Non-G ABCG5 band (Figure 2a), it is quite obvious that there is some contribution of STT3B-dependent post-translational mechanism in the regulation of ABCG5 N-glycosylation, which is consistent with the conclusion obtained from the data of pulse-chase analysis (Figure 2 f,g,h,i). On the other hand, N591 of ABCG5 seems to be in part co-translationally N-glycosylated because half of ABCG5 is originally produced as a Mono-G form (Figure 2f,i, 0 hr, Mono-G, 45.4%). However, STT3A knockdown does not increase deglycosylated forms (Mono-G, Non-G) of ABCG5 (Figure 2a). This may be due to possibilities that (1) basal expression level but not N-glycosylation status of ABCG5 is directly or indirectly regulated by STT3A, or (2) N-glycosylation of ABCG5 is actually regulated by STT3A, however, the stability of si-STT3A-induced non-G ABCG5 might be too low to visualize in the condition we used in the study. Further analysis would be needed to access how ABCG5 N-glycosylations at N584 and N591 sites are regulated, or especially which isoforms of STT3 proteins are involved.

Another point to be considered is whether HRD1 also affects the glycosylation of ABCG5 protein. At most, there is a very faint Non-G ABCG5 band after HRD1 overexpression (Supplementary Figure S7a, Figure 6g). Importantly, the faint Non-G ABCG5 band can be also observed by si-STT3B introduction (Figure 2a). Moreover, both WT- and C329S-HRD1 overexpression in the ABCG5/8 co-expression model seems to slightly increase the Non-G ABCG5 band as well as strongly increase the Non-G ABCG8 band (Figure 7c). These data suggest that HRD1 also inhibits ABCG5 N-glycosylation *via* the inhibition of STT3B-dependent N-glycosylation machinery although the inhibitory effect seemed to be very low. The specific action of HRD1 in the regulation of N-glycosylation of ABCG8, but not of ABCG5, and the fact that no change in mRNA expression of STT3A and STT3B was observed during HRD1 transfection condition (Supplementary Figure S9), support the idea that HRD1 does not regulate STT3B, but instead sequesters ABCG8.

In general, N-glycosylation of secretory or membrane glycoproteins in the ER has an important role in the ER quality control (ERQC) system^2^. Consistently, hypoglycosylation causes defects in glycoprotein folding, secretion, and function that sometimes contribute to the pathophysiological symptoms^21^. Until now, most of the research has focused on “glycosylation-dependent” ERQC system and characterized dynamic “glycan code” theories^2^,^5^. Although in previous studies mutational analysis of glycoproteins clearly demonstrated that Non-G form of glycoproteins is generally susceptible to ERAD^9^,^10^,^20^,^22^,^23^,^24^, factors that compel substrate glycoproteins to be degraded in a “non-glycosylation (Non-G)-dependent” manner have been unknown. The present study is first to show ABCG8 as one of the proteins that could be subjected to “Non-G-dependent” ERAD and to demonstrate that this is mediated by HRD1 E3 ubiquitin ligase through the suppression of STT3B-dependent N-glycosylation, regardless of its E3 ligase activity. Identification of the molecules that are critical for the “Non-G-dependent” ERAD is essential to further understand this unique ERQC system.

Overall, our data demonstrate that RMA1 and HRD1 are negative regulators of ABCG5/ABCG8 transporter (Figure 7e). What is the physiological relevance of RMA1- and HRD1-dependent ABCG5/8 regulation? Growing evidence supports the importance of expression levels and function of RMA1 and HRD1 in the physiological and pathophysiological conditions. RMA1 expression is associated with autophagic^25^ and anti-viral responses^26^, and disease progression such as degenerative myopathy^27^ and breast cancer^28^. On the other hand, HRD1 has been shown to be up-regulated during ER stress to protect the cells by eliminating misfolded proteins including mutant insulin and amyloid precursor protein^29^,^30^. Because there are no reports evaluating the correlation between the levels of RMA1 or HRD1 and the incidence of lipids-related diseases which could be caused by defects of ABCG5/8 transporter, epidemiological assessment may be beneficial to help to clarify the physiological relevance of our findings.

In summary, we have shown that both ABCG5 and ABCG8 proteins are post-translationally N-glycosylated. Among theses, ABCG8 is a novel protein that is post-translationally N-glycosylated *via* STT3B. Given the facts that the reports on STT3B-targeted substrate proteins are quite limited and this becomes a first example within multi-spanning membrane glycoproteins, our findings would provide new insights into fundamental mechanisms in the ERQC of other ABC transporters and/or membrane proteins. Moreover, among the results that showed suppression of ABCG5 and ABCG8 proteins by RMA1 and HRD1, the finding of E3 activity-independent negative regulation of post-translational ABCG8 glycosylation by HRD1 may open a gate to how Non-G-dependent ERAD pathway is physiologically and pathophysiologically regulated. Although our si-HRD1 approaches were only able to show the increase in the steady state expression of HM ABCG8 (Figure 5k) and the increase of mature forms of ABCG5 and ABCG8 proteins (Supplementary Figure S10), but not able to directly prove the obvious change of glycosylation state of ABCG8, due to an undetectable base line expression of Non-G ABCG8 protein at the experimental condition, our studies demonstrate a HRD1-dependent unique inhibitory mechanism of ABCG8 post-translational N-glycosylation, which results in a decreased ABCG8 protein expression, thereby suppressing full maturation of ABCG5/8 transporter.

## Methods

### DNA constructs

Cloning of human ABCG5 and ABCG8 cDNA (accession numbers NM022436 and NM022437, respectively) and introduction of Myc-tag and HA-tag sequences into the constructs were as previously described^31^. Human Myc-ABCG5 cDNA and human HA-ABCG8 cDNA were subcloned into pTRE-Shuttle2 vector. For the expression of ABCG5 and ABCG8, pTRE-Shuttle2 vectors containing Myc-ABCG5 and HA-ABCG8 were co-transfected with pTet-off vector (Takara-Clontech, Tokyo, Japan). Glycosylation-defective mutants N584Q-, N591Q-, N584Q/N591Q-ABCG5, N619Q-ABCG8, and E3-defective mutants C291S/C329S-HRD1, H260Q CHIP and C42S-RMA1 were generated using the QuikChange II site-directed mutagenesis kit (Stratagene, La Jolla, CA) according to previous reports^12^,^17^,^32^,^33^,^34^. Expression plasmids for WT CHIP and WT Rma1 were constructed as previously described^17^. Expression plasmids for WT HRD1 and C329S HRD1 were constructed as previously described^32^. Expression plasmids for WT GP78 and GP78 R2M were kindly provided by Dr. Allan M Weissman (National Cancer Institute, NIH)^35^.

### Reagents and antibodies

MG-132 was purchased from Calbiochem (San Diego, CA). Cycloheximide (CHX), digitonin, protease inhibitor cocktail and chloroquine were purchased from Sigma (St. Louis, MO). Endoglycosidase H (Endo H) and Peptide:N-Glycosidase F (PNGase F) were purchased from New England Biolabs (Ipswich, MA). Protein G Sepharose 4 Fast Flow was purchased from GE Healthcare (Waukesha, WI). The following antibodies were used in this study: Anti-myc monoclonal antibody (9E10), anti-HA probe monoclonal antibody (F7), anti-Rma1/RNF5 monoclonal antibody and non-immunized control IgG (Santa Cruz Biotechnology, Santa Cruz, CA), anti-myc polyclonal antibody (06-549; Upstate-Millipore, Charlottesville, VA), anti-HSC70 (clone 1B5; SPA-815; Stressgen Biotechnologies, Victoria, BC, Canada), anti-HRD1 (SYVN1; C-term; ABGENT, San Diego, CA), anti-HA polyclonal antibody (ab9143; Abcam Inc., Cambridge, MA), anti-ABCG8 polyclonal antibody (NB400-110; NOVUS Biologicals, Littleton, CO).

### Cell culture, transfection of plasmid DNA and siRNA

HEK293 cells were cultured in Dulbecco's modified Eagle's medium (DMEM, Wako) supplemented with 10% fetal bovine serum (FBS). Cells were maintained at 37°C in a humidified atmosphere of 5% CO_2_ and 95% air. Transient transfections of plasmid DNA were performed with Hilymax (Dojin Co. Ltd, Tokyo, Japan) by following the manufacturer's recommendations. Stealth™ RNAi targeting STT3A (HSS105598), STT3B (HSS176509) and negative Universal Control were purchased from Invitrogen (Carlsbad, CA). siRNAs targeting to human HRD1 (5′- CGTTCCTGGTACGCCGTCATT-3′) and GL2 luciferase (negative control) (5′-CGUACGCGGAAUACUUCGATT-3′) were synthesized and obtained from Sigma Genosys Aldrich (Japan). For siRNA transfection, siSTT3A and siSTT3B (100 nM) were transiently transfected into HEK293 by Lipofectamine RNAiMAX (Invitrogen) according to the manufacturer's instructions. Stealth RNAi Low GC Duplex was used as a negative control siRNA. To co-transfect siRNA and cDNA plasmids, plasmid transfection was performed after 24 hr of siRNA transfection, and then cells were cultured for further 48 hr. The knockdown efficiency was confirmed by quantitative real-time RT-PCR (Q-RT-PCR).

### Cell lysis, SDS-PAGE, Immunoblotting

Cells were washed twice with ice-cold PBS and lysed in RIPA Buffer (0.05 M Tris-HCl (pH 7.5),0.15 M NaCl,1% v/v Nonidet P-40,1% w/v Na deoxycholate,1% protease inhibitor cocktail) or 1% Triton Buffer (50 mM Tris–HCl (pH 7.5), 150 mM NaCl and 1% Triton-X-100, supplemented with 1% protease inhibitor cocktail (Sigma)). The lysates were cleared by centrifugation at 13,000 × *g* for 15 min. The protein concentration of the lysates was determined using bicinchoninic acid kit (Sigma), and equal amount of protein lysates were loaded and were separated by SDS-PAGE, immunoblotted with the indicated antibodies and visualized by chemiluminescence reagent SuperSignal (Thermo Fischer Scientific, San Jose, CA). In most of the data in main text, gels have been cropped for clarity; the bands were confirmed by the comparison with full-length gel images (Supplementary Figure S2–S4) and molecular weight.

### Quantitative real-time RT-PCR analysis

Total RNA was isolated from cells with RNAiso Plus (TaKaRa, Japan) according to the manufacturer's instruction. Q-RT-PCR analysis for the indicated nucleotides and internal control 18S ribosomal RNA (18S rRNA) were carried out with SYBR Green Master Mix (TaKaRa, Japan) following the manufacturer's instructions. The threshold cycle values for each gene amplification were normalized by subtracting the threshold cycle value calculated for 18S rRNA (internal control). The oligonucleotide primers used in the Q-RT-PCR are as follows: human STT3A (forward 5′-GTGTGGACCGTGAAGGTTCTC-3′ and reverse 5′-ATCCTGACCAGCCAATGTTCTG-3′), human STT3B (forward 5′-AATCCACCTGTGGAGGACAGC-3′ and reverse 5′-TGTGACCCAGGTACAGTGGAC-3′), human 18S rRNA (forward 5′-CGGCTACCACATCCAAGGAA-3′ and reverse 5′-GCTGGAATTACCGCGGCT-3′).

### Immunoprecipitation

Confluent (90–100%) cells grown on 60-mm dishes were lysed by gentle rotation in 500 μl of digitonin buffer (50 mM Tris–HCl, 150 mM NaCl, 1% digitonin, and 1% protease inhibitor cocktail) for 2 hr at 4°C and centrifuged at 15,000 × *g* for 10 min at 4°C. The supernatant was gently rotated for 2 hr at 4°C with primary antibodies, followed by the additional incubation with protein G-Sepharose® 4 Fast Flow for 1 hr at 4°C. Immune complexes were precipitated and washed three times with 1 ml of digitonin wash buffer (50 mM Tris–HCl, 150 mM NaCl, 0.1% digitonin, and 1% protease inhibitor cocktail). Immunoprecipitated proteins were eluted for 30 min at 37°C with 2× concentrated loading buffer and analyzed by Immunoblotting.

### Analysis of protein stability and enzymatic digestion of N-glycosylation

For evaluation of protein stability in the cells, CHX chase experiment was performed as previously described. Briefly, plasmids- and/or siRNA-transfected cells were treated with CHX (50 μM) for the time periods indicated and lysed and analyzed by immunoblotting. For the Glycosidase digestion, cell lysates were incubated with Endo H (500 U) and PNGase F (500 U) for 2 hr at 37°C. The density of the bands was quantified using Image Gauge software (version 4.23; Fujifilm).

### Pulse-chase experiments

Cells were incubated with methionine/cysteine-free DMEM (Invitrogen) for 30 min. Cells were then pulse-labeled with 100 μCi/ml EXPRE35S35S protein labeling Mix (PerkinElmer Life & Analytical Sciences, MA) for 5, 10, 15 or 30 min and chased in fresh complete medium containing 5 mM methionine and cysteine for the indicated times. Cells were lysed in 1% Triton Buffer containing 1% protease inhibitor cocktail and subjected to immunoprecipitation with anti-HA or c-Myc antibody. Radioactivity was analyzed by BAS imaging plate scanner (BAS-2000; Fujifilm, Japan) and quantified using Image Gauge software (version 3.4; Fujifilm, Japan).

### Statistical analysis

For quantitative analysis, most of the result represents the mean ± SEM performed in triplicate and the data were analyzed by either Student's *t*-test or one-way ANOVA with Dunnett's test (JMP software, SAS Institute) as indicated in each figure legend.

## Author Contributions

S.S., T.Sh., T.Sa., M.K. and T.T. performed cell culture, biochemical and molecular biological experiments. S.S., T.Sa., M.K., T.T., D.M.C. and H.S. generated plasmids and cell lines. S.S., T.Sh., T.Sa., M.A.S., H.S. and H.K. designed experiments and interpreted the data. S.S., T.Sh. and M.A.S. wrote the manuscript. T.Sh. and H.K. supervised the project.

## Supplementary Material

Supplementary Information SUPPLEMENTARY FIGURES
