# Supplementary material for: Inhibition of post-translational N-glycosylation by HRD1 that controls the fate of ABCG5/8 transporter
Source: Sci Rep. 2014 Mar 3;4:4258. doi: 10.1038/srep04258 (PMC3939451; doi:10.1038/srep04258)
Supplement: Supplementary Information — SUPPLEMENTARY FIGURES [file srep04258-s1.pdf]

Supplemental information

Inhibition of post-translational N-glycosylation by HRD1 that controls the fate of ABCG5/8 transporter

Shingo Suzuki, Tsuyoshi Shuto, Takashi Sato, Masayuki Kaneko, Tappei Takada, Mary Ann Suico, Douglas M. Cyr, Hiroshi Suzuki, Hirofumi Kai

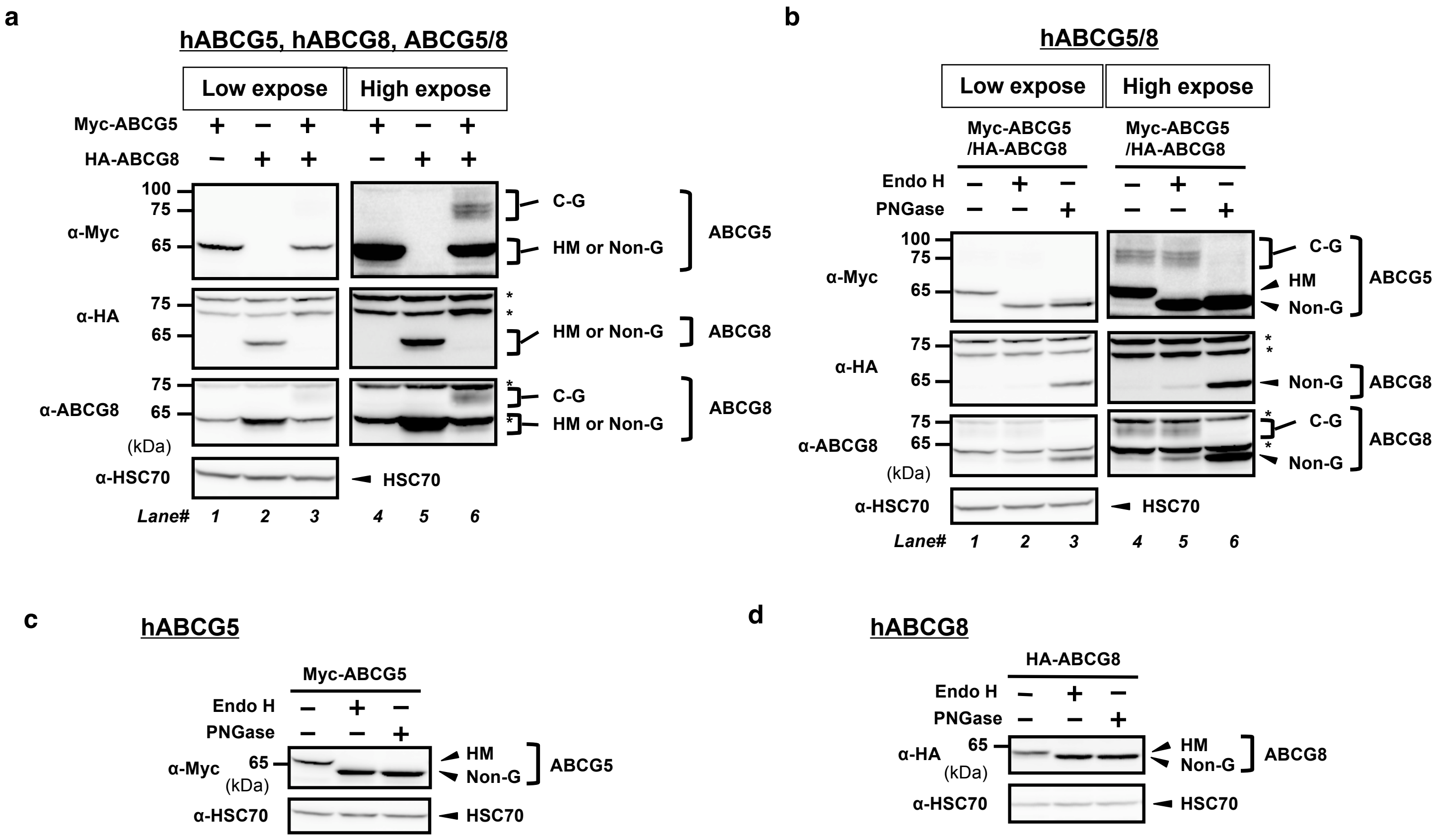

Supplementary Figure S1. Determination of band patterns of monomeric and heterodimeric human ABCG5 and ABCG8. Steady-state expression of monomeric Myc-tagged ABCG5 (Myc-ABCG5), HA-tagged ABCG8 (HA-ABCG8) and heterodimeric Myc-ABCG5/HA-ABCG8 was analyzed by immunoblotting to detect ABCG5 and ABCG8 proteins (**a**). Cell lysates were further digested with Endo H (500U) or PNGase F (500U), followed by immunoblotting to detect ABCG5/8 heterodimer (**b**), ABCG5 monomer (**c**) and ABCG8 monomer (**d**). * indicates non-specific band. Complex-glycosylated, high-mannose and non-glycosylated forms of ABCG5 and ABCG8 proteins are represented as C-G, HM and Non-G, respectively (scheme is indicated in Figure 1f of main text). Gels have been cropped for clarity; full-length gels of Supplementary Figure S1a and S1b are presented in Supplementary Figure S2 and S3, respectively. The bands of Supplementary Figure S1c and S1d were confirmed by the comparison with full-length gel images (Supplementary Figure S4) and molecular weight.

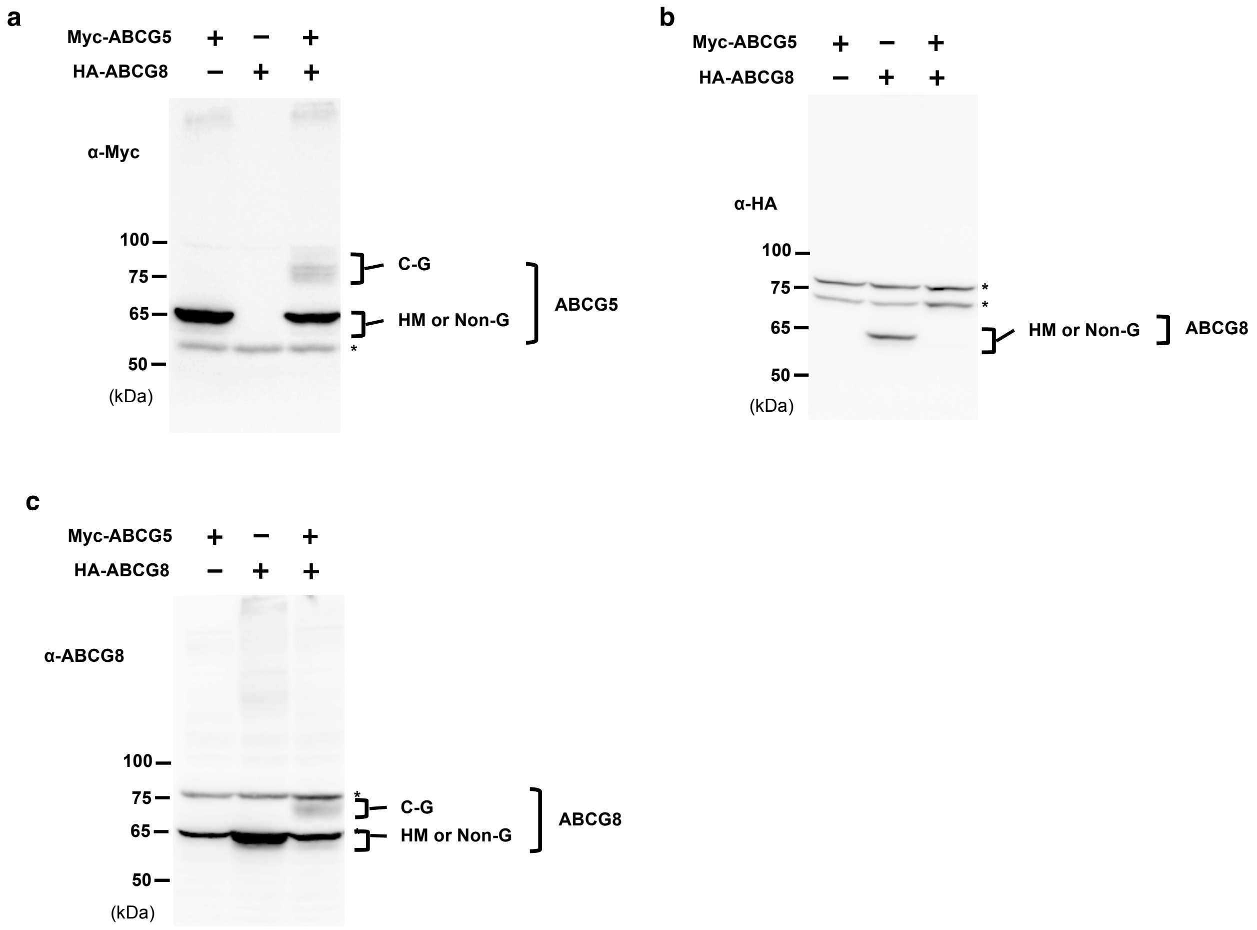

Supplementary Figure S2. Full-length gel images of Supplementary Figure S1a. Western blotting data using anti-myc (**a**), anti-HA (**b**) and anti-ABCG8 (**c**) antibodies Molecular weight was indicated based on the dual color marker (Bio-Rad). * indicates non-specific band. Complex-glycosylated, high-mannose and non-glycosylated forms of ABCG5 and ABCG8 proteins are represented as C-G, HM and Non-G, respectively (scheme is indicated in Figure 1f of main text).

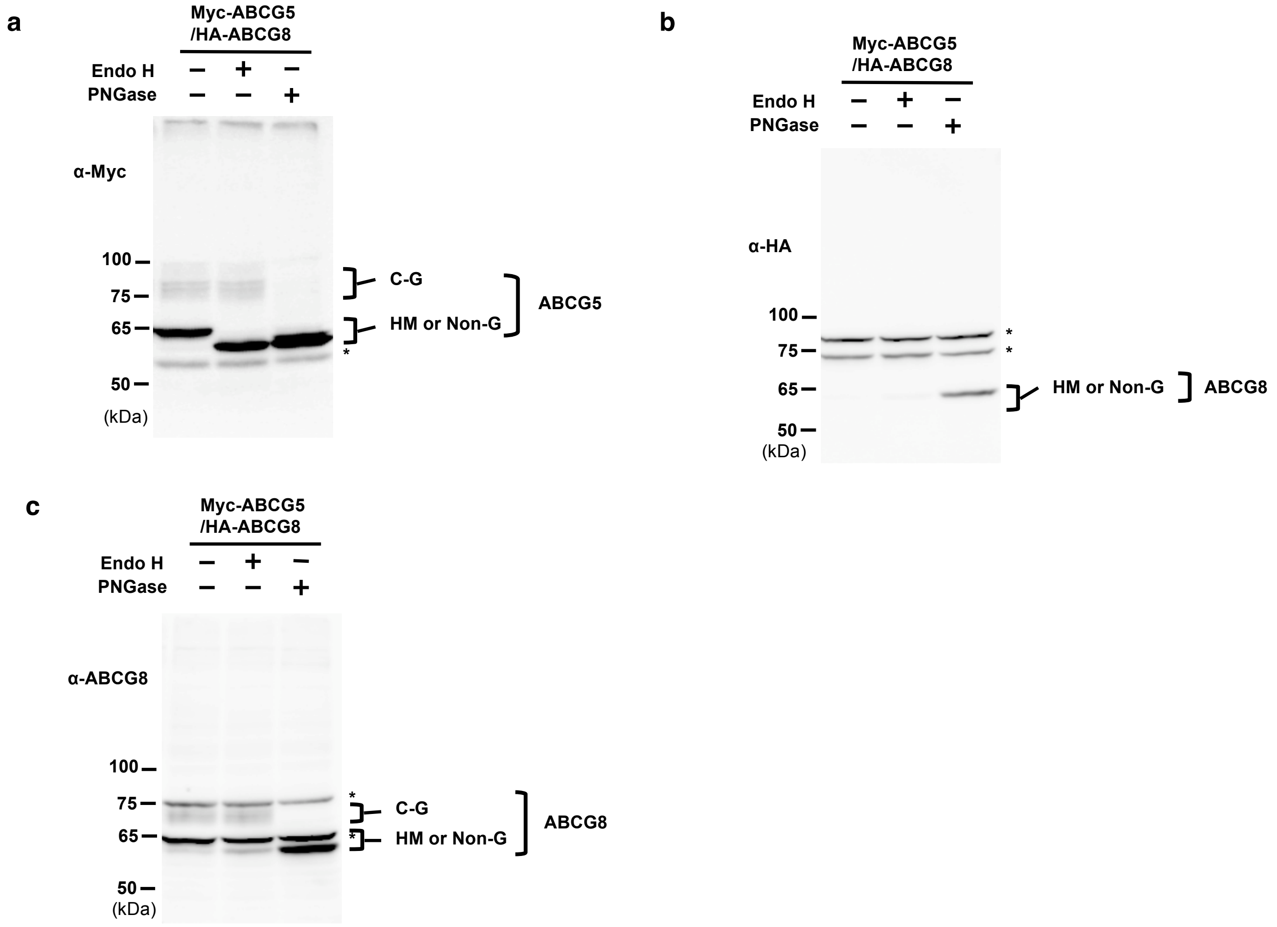

Supplementary Figure S3. Full-length gel images of Supplementary Figure S1b. Western blotting data using anti-myc (**a**), anti-HA (**b**) and anti-ABCG8 (**c**) antibodies Molecular weight was indicated based on the dual color marker (Bio-Rad). * indicates non-specific band. Complex-glycosylated, high-mannose and non-glycosylated forms of ABCG5 and ABCG8 proteins are represented as C-G, HM and Non-G, respectively (scheme is indicated in Figure 1f of main text).

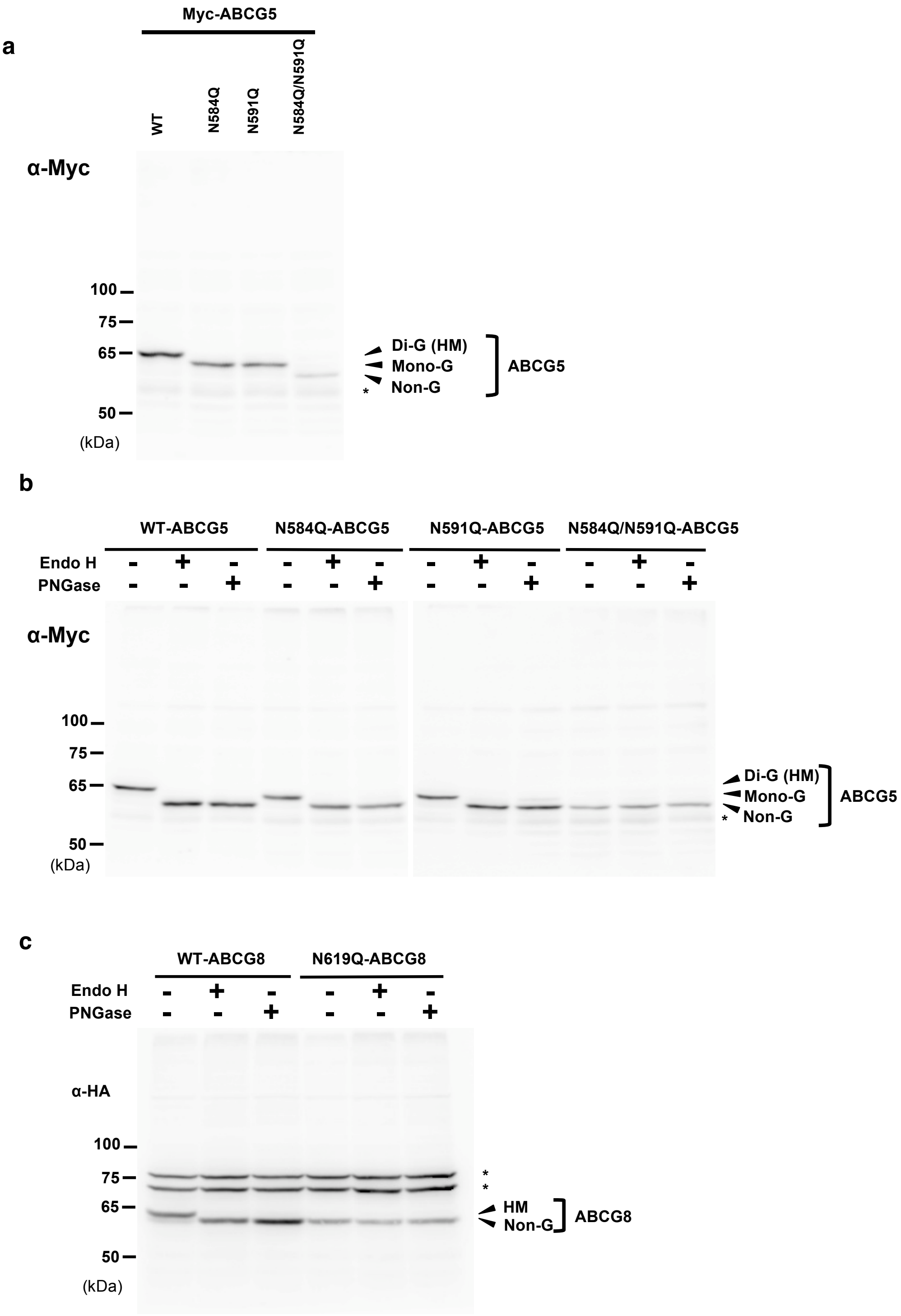

Supplementary Figure S4. Full-length gel images of Figure 1b, 1c and 1e. Western blotting data using anti-myc (**a,b**) and anti-HA (**c**) antibodies. Molecular weight was indicated based on the dual color marker (Bio-Rad). * indicates non-specific band. Complex-glycosylated, high-mannose, di-glycosylated, mono-glycosylated and non-glycosylated forms of ABCG5 and ABCG8 proteins are represented as C-G, HM, Di-G, Mono-G and Non-G, respectively (scheme is indicated in Figure 1f of main text).

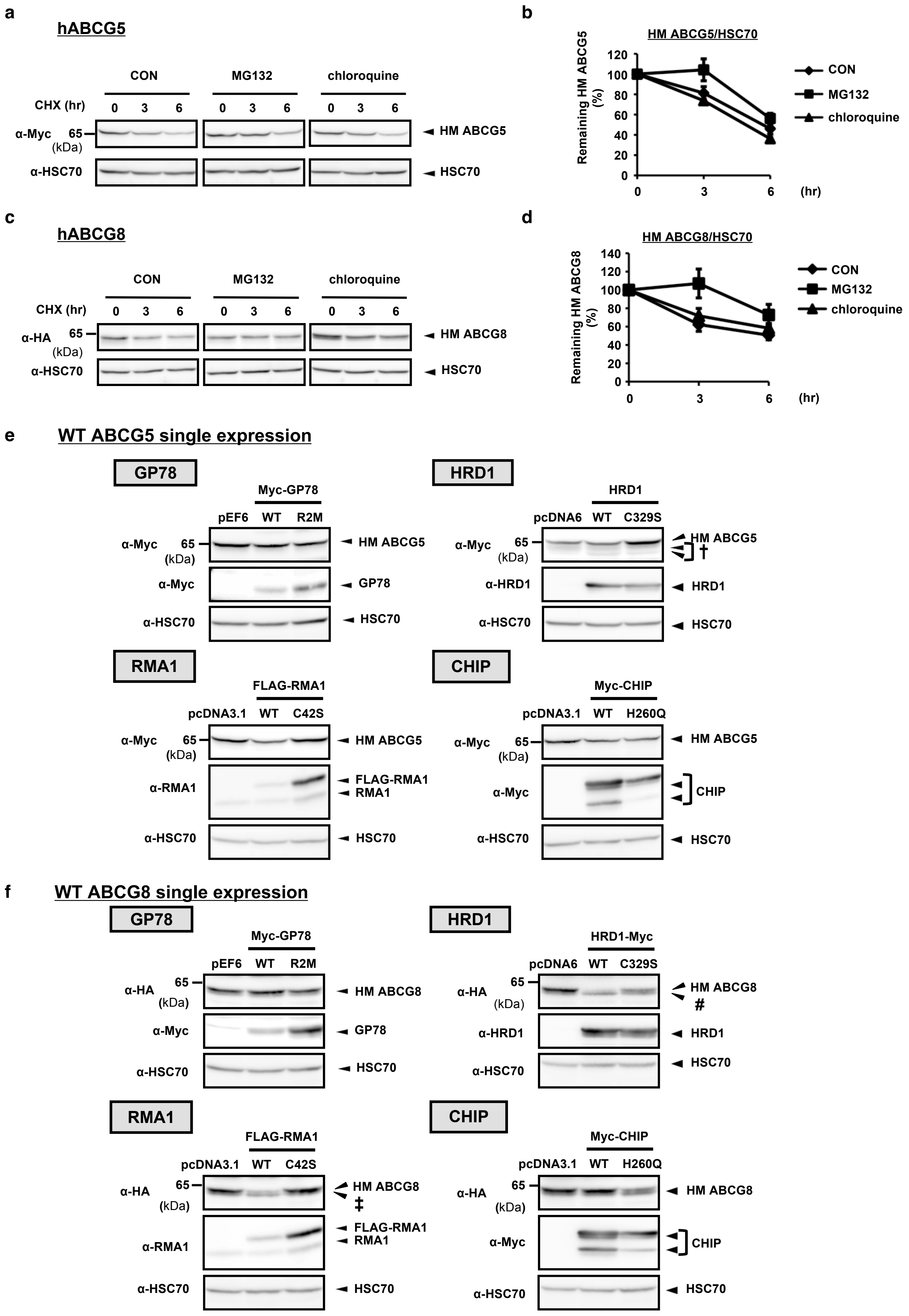
a
hABCG5
CON
MG132
chloroquine
CHX (hr)
0 3 6
α-Myc
65
(kDa)
HM ABCG5
α-HSC70
HSC70
b
HM ABCG5/HSC70
Remaining HM ABCG5 (%)
0
20
40
60
80
100
120
0 3 6 (hr)
CON
MG132
chloroquine
c
hABCG8
α-HA
HM ABCG8
d
HM ABCG8/HSC70
Remaining HM ABCG8 (%)
140
e
WT ABCG5 single expression
GP78
Myc-GP78
pEF6 WT R2M
GP78
HRD1
pcDNA6 WT C329S
†
α-HRD1
RMA1
FLAG-RMA1
pcDNA3.1 WT C42S
α-RMA1
FLAG-RMA1
RMA1
CHIP
Myc-CHIP
pcDNA3.1 WT H260Q
f
WT ABCG8 single expression
HRD1-Myc
#
‡

Supplementary Figure S5. Identification of degradation pathway of ABCG5/8 proteins and effect of ER-localized E3 ubiquitin ligases and their E3 dominant-negative mutants on the expression of ABCG5 and ABCG8 proteins. (**a-d**) Stability of Myc-ABCG5 (**a**,**b**) or HA-ABCG8 (**c**,**d**) in HEK293 cells treated with or without proteasome inhibitor, MG132 (30 μM) or lysosome inhibitor, chloroquine diphosphate (150 μM) was determined by cycloheximide (CHX) (200 μM) chase experiments. S-G forms of ABCG5 (**b**) and ABCG8 (**d**) were quantified and normalized by HSC70 expression. Data are presented as the percentage of the amount detected at 0 hr (n=3). (**e**,**f**) Steady-state expression of HEK293 cells co-transfected with Myc-ABCG5 (**a**) or HA-ABCG8 (**b**) and empty vector, epitope-tagged WT or dominant negative form of E3 ligases (GP78, HRD1, RMA1, CHIP). Cells were lysed in RIPA buffer 48 hr after transfection. Cell lysates were subjected to immunoblotting with indicated antibodies. HSC70 was used as internal control. †, ‡, # indicate the band of lower molecular weight (LMW). Gels have been cropped for clarity; the bands were confirmed by the comparison with full-length gel images (Supplementary Figure S4) and molecular weight.

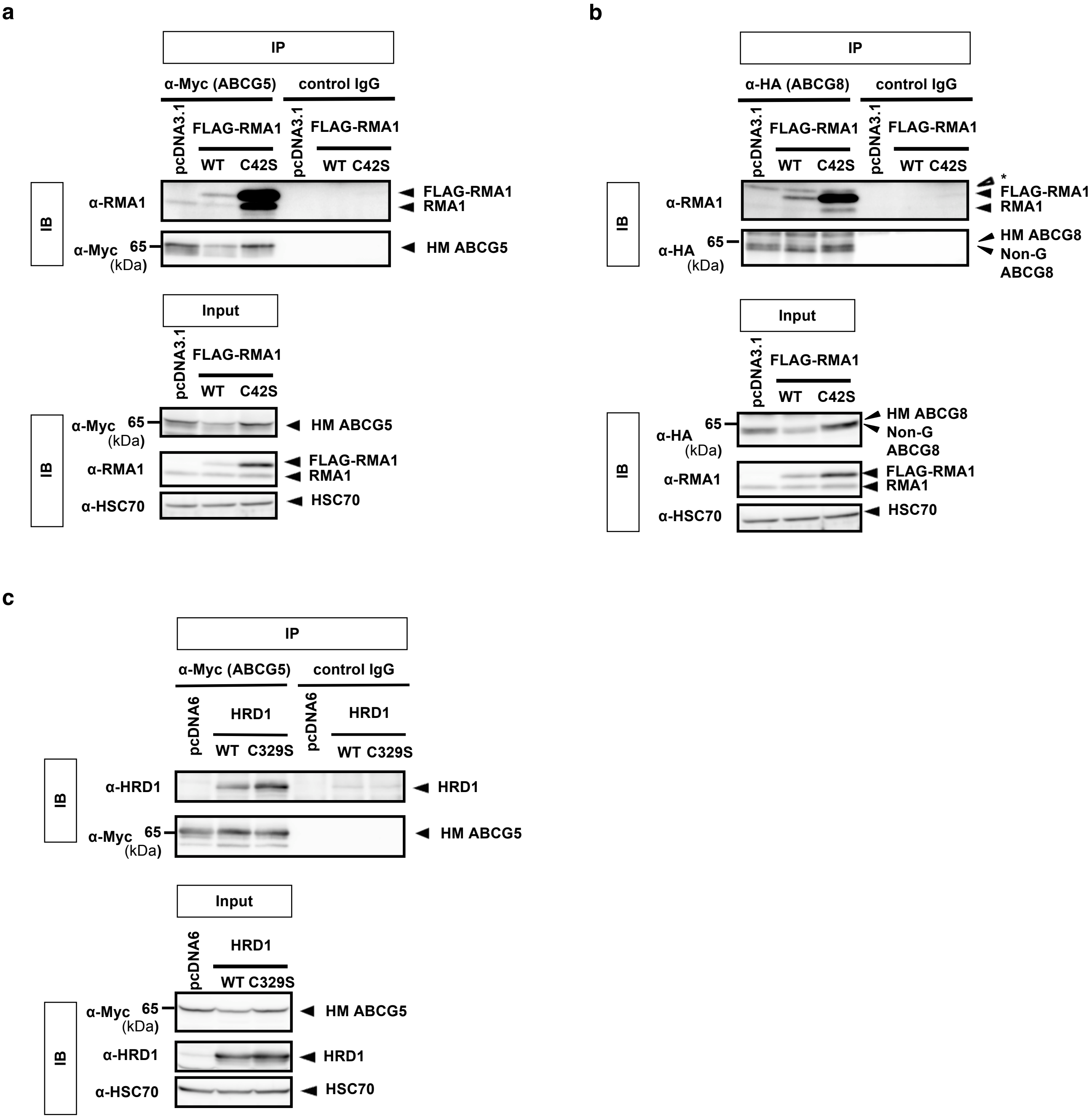

Supplementary Figure S6. S-G forms of ABCG5 and/or ABCG8 proteins interact with RMA1 and/or HRD1 proteins. (**a**,**b**) ABCG5 (**a**)- or ABCG8 (**b**)-expressing HEK293 cells were transfected with expression vectors that encode Flag-tagged WT or C42S RMA1. ABCG5 or ABCG8 proteins were immunoprecipitated by using anti-Myc and anti-HA antibodies, respectively, or control IgG, and immunoprecipitants were analyzed by immunoblotting (upper panel). Input protein was used as positive control (lower panel). (**c**) ABCG5-expressing HEK293 cells were transfected with expression vectors that encode WT or C329S HRD1. ABCG5 protein was immunoprecipitated by using anti-Myc antibody or control IgG, and immunoprecipitants were analyzed by immunoblotting (upper panel). Input protein was used as positive control (lower panel). Gels have been cropped for clarity; the bands were confirmed by the comparison with full-length gel images (Supplementary Figure S4) and molecular weight.

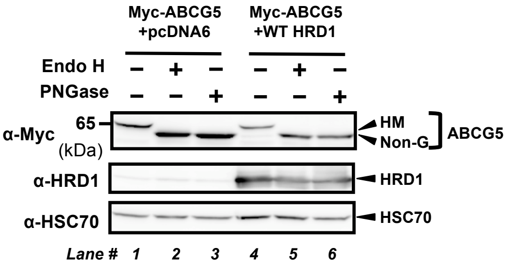

Supplementary Figure S7. Characterization of LMW ABCG5 protein induced by HRD1. Cell lysates from Myc-ABCG5-expressing HEK293 cells transfected with empty vector or an expression vector that encodes WT HRD1 were further digested with EndoH or PNGase F, and analyzed by immunoblotting. Gels have been cropped for clarity; the bands were confirmed by the comparison with full-length gel images (Supplementary Figure S4) and molecular weight.

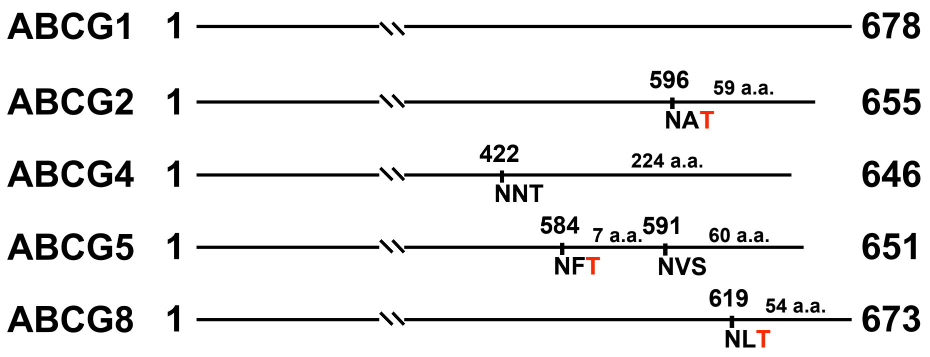

Supplementary Figure S8. Sequon analysis in ABCG subfamily. Diagram of ABCG subfamily proteins showing the glycosylation sites. Amino acids Asn, Thr, Ser, Ala, Phe, Val and Leu are represented as N, T, S, A, F, V and L. Length of a.a. sequence from C-teminus to sequons is indicated. Thr site located at the boundary, which could be a candidate site for post-translational N-glycosylation by STT3B (Shrimal S., et al. J Cell Biol. 201:81-95. 2013), is shown in red.

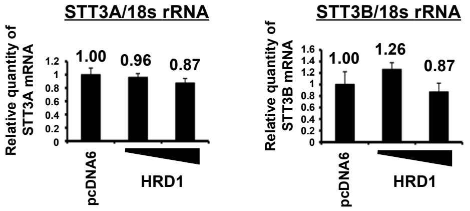

Supplementary Figure S9. HRD1 does not affect mRNA expression of STT3A and STT3B. Expression levels of STT3A and STT3B genes were analyzed by Q-RT-PCR in HRD1-transfected HEK293 cells. Human 18s ribosmal RNA was used as internal control (n=3).

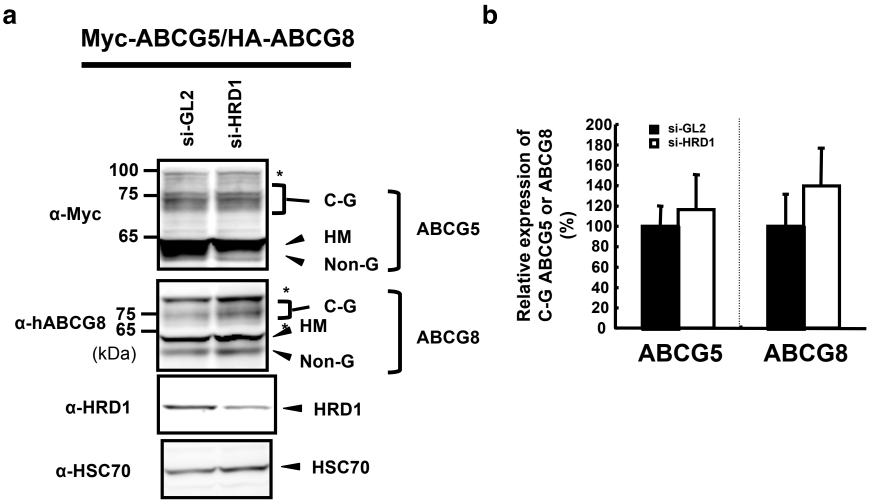

Supplementary Figure S10. Effect of si-HRD1 on the expression of matured ABCG5 and ABCG8 proteins. Steady-state expression of C-G forms of ABCG5 and ABCG8 proteins in HEK293 cells transfected with si-HRD1 was analyzed by immunoblotting.* indicates non-specific band (**a**). Mature C-G forms of ABCG5 and ABCG8 protein bands after transfection with si-GL2 or si-HRD1were calculated and data are presented as the percentage of the amount detected in si-GL2-transfected control (n=3) (**b**). Gels have been cropped for clarity; the bands were confirmed by the comparison with full-length gel images (Supplementary Figure S2 and S3) and molecular weight.
